# Effect of atmospheric cold plasma on the quality of fish meat from different parts of larger yellow croaker during refrigeration

**DOI:** 10.3389/fnut.2026.1750328

**Published:** 2026-01-27

**Authors:** Chenyi Yang, Jiaxi Cai, Shanggui Deng, Mengyuan Xu, Huiqi Dai, Sitong Shan, Yuanpei Gao, Ruizhi Yang, Hao Lan

**Affiliations:** 1 School of Food and Science, Zhejiang Pharmaceutical University, Ningbo, China; 2 School of Food and Pharmacy, Zhejiang Ocean University, Zhoushan, China

**Keywords:** atmospheric cold plasma, flavor, large yellow croaker, preserve freshness, quality

## Abstract

In this study, the effects of atmospheric cold plasma (ACP) as a non-thermal sterilization method on the quality of different muscle tissues parts in Large yellow croaker (*Larimichthys crocea*) during refrigerated storage were systematically investigated. The results of this study demonstrate that atmospheric cold plasma treatment (ACP, 40 kV, 90 s) significantly inhibited the accumulation of TVB-N and TBARS (*p* < 0.05) in fish samples while effectively mitigating the decline in water-holding capacity. Compared with the control group, the total sulfhydryl content in dorsal and ventral muscles increased significantly by 20 and 45%, respectively, and Ca^2+^-ATPase activity was also significantly enhanced by 14 and 20% (*p* < 0.05) after treatment. Furthermore, ACP treatment significantly maintained the color parameters (L*, a*, b*) and textural properties (hardness, elasticity, etc.) of fish meat (*p* < 0.05). ACP has an inhibitory effect on post-oxidation. GC–MS analysis detected 60 volatile compounds, with significantly reduced aldehyde levels in the treated group, demonstrating effective inhibition of undesirable flavor formation. These findings collectively indicate that ACP treatment effectively delays quality deterioration in fish during refrigerated storage through multiple synergistic mechanisms.

## Graphical abstract

**Figure:**
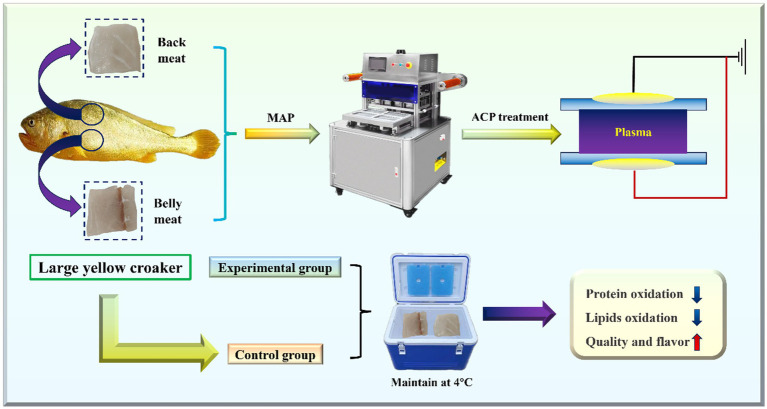

## Highlights

- Dynamic monitoring was conducted on quality variations across various muscle regions of refrigerated large yellow croaker after ACP treatment.
- The study clarifies the relationship between water-holding capacity and moisture migration in large yellow croaker muscle subjected to ACP treatment.
- ACP exhibited a dual-phase effect: it initially accelerated lipid oxidation in fish flesh, while subsequently demonstrated significant antioxidative protection.
- ACP an effectively inhibit the formation of undesirable flavor compounds in Large yellow croaker fish flesh.

## 1 Introduction

As a significant economic marine fish in China, large yellow croaker (*Larimichthys crocea*) occupies a prominent position in fishery resources. Its tender meat possesses a unique flavor and is rich in high-quality protein, unsaturated fatty acids, and other essential nutrients, which contribute to its considerable culinary and economic value (1, 2). However, due to the high moisture content and intense enzymatic activity in the muscle tissue, the fish is prone to spoilage and deterioration, which significantly hampering its commercial distribution. While conventional preservation techniques can extend shelf life, they often introduce drawbacks, including prolonged processing times, residual chemicals, and compromised product quality (3). Although low-temperature storage with chilled preservation is currently the primary method employed to mitigate fish quality deterioration, temperature fluctuations during logistics and sales can jeopardize tissue integrity and freshness parameters, ultimately diminishing both sensory attributes and market value (4). Currently, researchers urgently need to explore a novel preservation method to mitigate these issues.

Atmospheric cold plasma (ACP) technology has shown promising potential for quality maintenance of perishable aquatic commodities due to its high efficiency, low energy consumption, and environmentally friendly characteristics (5). Scholars such as Baier et al. (6). Have demonstrated that ACP, as a non-thermal sterilization technology, can effectively inhibit foodborne microorganisms while preserving the organoleptic and nutritional quality of food. However, ACP inactivates microorganisms primarily through high-voltage electric fields that disrupt cell membrane integrity, while simultaneously generating ROS (reactive oxygen species) and RNS (reactive nitrogen species) which induce lipid peroxidation, protein denaturation, and DNA damage (7). For instance, the total number of microorganisms in ACP-treated tilapia filets was notably reduced, resulting in an extended shelf life (8). In the preservation of red shrimp, ACP treatment not only effectively suppresses TVB-N accumulation but also better maintains shrimp color stability (9). Although ACP has demonstrated efficacy in meat preservation, limited research has been conducted on its effects on large yellow croaker meat. Given the distinct characteristics of different fish parts, a comprehensive investigation into the quality changes of various muscle sections during storage is warranted. Despite growing interest in ACP technology, its effects on the post-processing quality evolution of commercially important large yellow croaker during storage remain underexplored.

Herein, we conducted a comprehensive assessment of changes in key quality indicators across different sections of large yellow croaker flesh following ACP treatment. This encompassed determinations of TVB-N, pH, and TBA; textural and color difference measurements; water migration analysis; sensory evaluation; Ca^2+^-ATPase activity assays; protein content quantification; thiol group determination; volatile flavor compound composition; and total fatty acid analysis. This study aims to provide a theoretical foundation and technical support for developing efficient and safe novel preservation technologies for large yellow croaker fish.

## 2 Materials and methods

### 2.1 Materials

Iced Fresh Large yellow croaker (*Larimichthys crocea*) from seafood market in Zhoushan, China. All chemicals and reagents were analytical grade.

### 2.2 Sample preparation and pretreatment

The experimental large yellow croakers were procured from Zhoushan International Seafood Market (Zhoushan, China). All specimens were purchased from the same batch, totaling 40 individuals. Selection criteria were: average weight (500 ± 50) g, intact appearance, bright coloration, and absence of mechanical damage. All specimens underwent immediate subsequent processing and analysis upon arrival. All biochemical and physical property tests in this study were conducted using these 40 fish samples from the same batch to ensure consistency of results. The fish were placed in a foam box with ice to maintain freshness. After removing the head, tail, guts, and skin, Muscle sections from both the abdominal muscle and dorsal muscle regions of the large yellow croaker were dissected and thoroughly rinsed with ultrapure water，then carefully dried using lint-free laboratory wipes. After that, the musle were placed in a sealed bag and stored at 4 °C for future use.

Samples were randomly separated into two groups. The ACP equipment employed was the Phenix BK130/36 ACP Processor (Phenix Technologies, United States). The ACP system comprised a Dielectric Barrier Discharge (DBD) reactor, utilizing high-purity (≥99.99%) ambient air as the working gas. No additional gas sources were introduced, with a flow rate of 2.0 ± 0.1 L/min. The electrode-sample distance was maintained at 18 mm. The polypropylene sample holder measured 135 mm × 210 mm × 35 mm. Most components were commercially available, while the reaction chamber was custom-fabricated to meet experimental requirements. The treatment batch was ACP-treated at 40 kV for 90 s in a sealed box. After treatment, the fish were transferred to a sealed bag. The second batch remained untreated. All samples were stored in temperature-controlled refrigeration (4.0 ± 0.2 °C). Systematic sampling was conducted on days 0, 2, 4, 6, and 8 of refrigerated storage to monitor temporal changes in quality parameters.

### 2.3 Determination of total volatile base nitrogen content

Based on the operational protocol defined by (10), a 10.00 g sample of minced large yellow croaker was placed in a distillation tube. The mixture, post-addition of 75 mL deionized water, was left undisturbed for a 30-min duration, followed by the addition of 1 g of magnesium oxide powder. The mixture was then distilled for 6 min using a Kjeldahl nitrogen analyzer (KDN-19A, Shanghai). The distillate was trapped in 30 mL of 2% boric acid (with 1 mL mixed indicator) and endpoint-titrated using 0.01 M HCl for TVB-N quantification.

### 2.4 Determination of pH value

Experiments were conducted with slight modifications based on the methodology of Cheng et al. (11). A precise weight of 2.00 g of fish meat was taken and stirred. The sample was homogenized with 18 mL ultrapure water in a 50 mL centrifuge tube (11,180 × g), subsequently, the mixture was placed in a centrifuge (5424R, Nanjing Eruoda) and centrifuged at 1,000 × g for 10 min at 4 °C. The supernatant was collected and aliquoted for subsequent analysis. The Ph of raw homogenate was determined through three independent measurements using a calibrated pH meter (S-3C, shanghai Hongyi), with electrodes rinsed with deionized water between measurements.

### 2.5 Determination of water holding capacity

Approximately 2.00 g of the sample was finely chopped, accurately weighed (W₁), and placed in a centrifuge tube lined with three pieces of filter paper. Following centrifugation (6,800 × g, 10 min, 4 °C), the sample was subjected to reweighing (W₂). WHC was calculated using the following Equation (1) (12):


$$\mathrm{WHC}(\%)=\frac{W_{2}}{W_{1}}\times 100$$ (1)

### 2.6 Color

The dorsal and ventral muscle tissues of large yellow croaker were uniformly processed into standardized geometric specimens measuring 2 cm (length) × 3 cm (width) × 2 cm (height). Measurements were conducted using a colorimeter (DS200), with six measurement points selected for each sample to ensure accurate data collection. The following formula (2) was employed to calculate the whiteness.


$$\text{Whiteness}=100-\sqrt{{\left(100-L^{*}\right)}^{2}+a^{*2}+b^{*2}}$$ (2)

In the parameter, the L* value indicates the luminance value, the a* represents the redness value, b* reflects the yellowness value.

### 2.7 Determination of texture profile analysis

The samples were cut into squares prisms measuring 2 × 3 × 2 cm, then washed and blotted dried with kitchen paper. The test was conducted using the TMS-Touch Texture Analyzer (FTC, United States): a deformation rate of 1 mm/s (approach/retract), a morphology of 50%, a residence time between of 1 s between two compressions, and a P/0.5-transparent spherical probe. Each sample was tested for six times, record the values for the parameters Hardness, Adhesiveness, Springiness, Cohesiveness, Chewiness, and Resilience, and the average was recorded (13).

### 2.8 Sensory assessment

This experimental protocol is adapted from the 2019 study by Chen et al. with minor methodological adjustments (14). Fifty healthy university students with no history of food allergies were recruited (equal numbers of males and females, aged 20–25 years). All participants were regular fish consumers (consuming fish ≥twice monthly) and possessed a fundamental interest in sensory evaluation. The experiment utilized a hedonic scale approach, requiring panelists to rate the samples on a 1–10 scale (1 indicating extreme dislike, 10 indicating extreme preference). The evaluation covered five key dimensions: color, odor, texture, appearance, and overall acceptability, under standardized lighting to avoid color distortion. Physical partitions were used between panelists during the evaluation to ensure independent assessments. Samples were maintained at a constant 4 °C throughout the process to preserve their initial quality. The final data were visualized using radar charts to intuitively compare differences in sensory indicators across treatment groups. The sensory experiment was approved on August 20, 2025, with the ethical review approval number: ZYLL202508010.

### 2.9 Low-field nuclear magnetic resonance

Three Scans were conducted on a sample with dimensions of 2 × 2 × 1 cm using a variable temperature MRI analyzer (NMI20-040 V-I, Suzhou Newmax) to detect the transverse relaxation time T2. The following parameter settings were employed: SW = 200 kHz, RFD = 0.08 ms, SF = 20 MHz, RG1 = 20 dB, DRG1 = 3, P1 = 8.40 μs, P2 = 14 μs, NECH = 2,500, TE = 0.8 ms, DR = 1, TW = 3,000 ms, and NS = 16.

The Niumag NMR Imaging System V 3.0 software was utilized to scan and obtain proton density-weighted pseudo-color images of the large yellow croaker, facilitating the assessment of water distribution and migration within the species (15).

### 2.10 Determination of thiobarbituric acid reactive substances

This protocol was adapted with minor modifications from the method established by Li et al. (16). Transfer exactly 5.00 g of sample to a 100 mL flask, followed by the quantitative addition of 50.0 mL TCA solution using a pipette, shaken well, sealed with a stopper, and then placed on a shaker to shake at 50 °C for 30 min, then remove and cool to ambient temperature (25 ± 2 °C), followed by filtration through double-layer filter paper. The primary filtrate was discarded, and the secondary filtrate was set aside for later use, absorbance was measured using a UV–visible spectrophotometer (Evolution™ Pro, Thermo Fisher Scientific, United States).

Next, 5 mL of the resultant solution was combined with an equivalent volume of TBA solution in a colorimetric tube through calibrated pipetting. In a separate colorimetric tube, 5 mL of the TCA mixture and 5 mL of TBA solution were combined. The tubes were capped, mixed thoroughly, followed by incubation in a precisely controlled water bath maintained at 90.0 ± 0.5 °C for 30 min Upon reaction completion, the samples were equilibrated to ambient temperature. After performing instrument blank correction using the reference solution, absorbance measurements were conducted at 532 nm with 1 nm spectral bandwidth for both test samples and standard calibrants.

### 2.11 Extraction of myofibrillar protein

Based on the method described by Li et al. in 2021, myofibrillar proteins were extracted with slight procedural adaptations (17). Freshly minced large yellow croaker muscle (5.00 g) was homogenized with 20 mL of ice-cold Tris–HCl buffer A (0.02 mol/L, pH 7.5) in a 50 mL polypropylene centrifuge tube using a high-speed homogenizer (FJ-200, shanghai Fluko) for 1 min at a speed of 11,180 × g. Following homogenization, the sample was pelleted by refrigerated centrifugation at 6,000 × g for 10 min (4 °C), followed by careful supernatant removal. The aforementioned washing procedure was repeated twice, resulting in a total of three washing cycles.

Then the supernatant was discarded, the precipitate was retained, to ensure complete protein extraction, 20 mL of Tris–HCl buffer B (0.02 mol/L, pH 7.5, 0.6 M NaCl) was incorporated, with subsequent homogenization (11,180 × g, 1 min) and centrifugation (3,000 × g, 20 min, 4 °C) to isolate target fractions. The resulting supernatant was the extracted myofibrillar protein.

### 2.12 Total sulfhydryl content and Ca^2+^-ATPase activity

The determination of total sulfhydryl groups in membrane proteins (MP) of both treated and control samples was conducted according to the description provided in the Total Sulfhydryl Groups Kit. Additionally, the ultra-trace ATPase (UTA) content of the membrane proteins in treated and control samples was assessed by following the guidelines outlined in the UTA (Ca^2+^-ATPase) kit.

### 2.13 Fatty acid content determination

The sample was hydrolyzed with pyrogallic acid, hydrochloric acid and ethanol (75 °C, 40 min), followed by triple solvent extraction to isolate lipids. After saponification and methylation, the fatty acid methyl esters were analyzed by GC (7890A, Agilent) using a TG-FAME column (18).

### 2.14 Analysis of volatile substances

The study employed gas chromatography–mass spectrometry (Agilent 6,890 N-G5795B) with headspace solid-phase microextraction for sample analysis. A sample weighing 5 g was placed into a 20 mL headspace vial, followed by the addition of 2-octanol (the internal standard) and saturated sodium chloride was added, then heated at 80 °C for 30 min. The sample was then injected into the headspace and heated for another 30 min, and finally desorbed in the injection port at 250 °C (18).

### 2.15 Data analysis

The experiments were all performed three times in parallel. Statistical analysis was conducted using IBM SPSS 22.0 software (SPSS Inc., Chicago, United States). To identify significant differences among the samples, we applied analysis of variance (ANOVA) along with Duncan’s multiple range test (*p* < 0.05). PCA analyzes were analyzed using Rstudio software.

## 3 Results and discussion

### 3.1 TVB-N and PH

**Figure 1:**
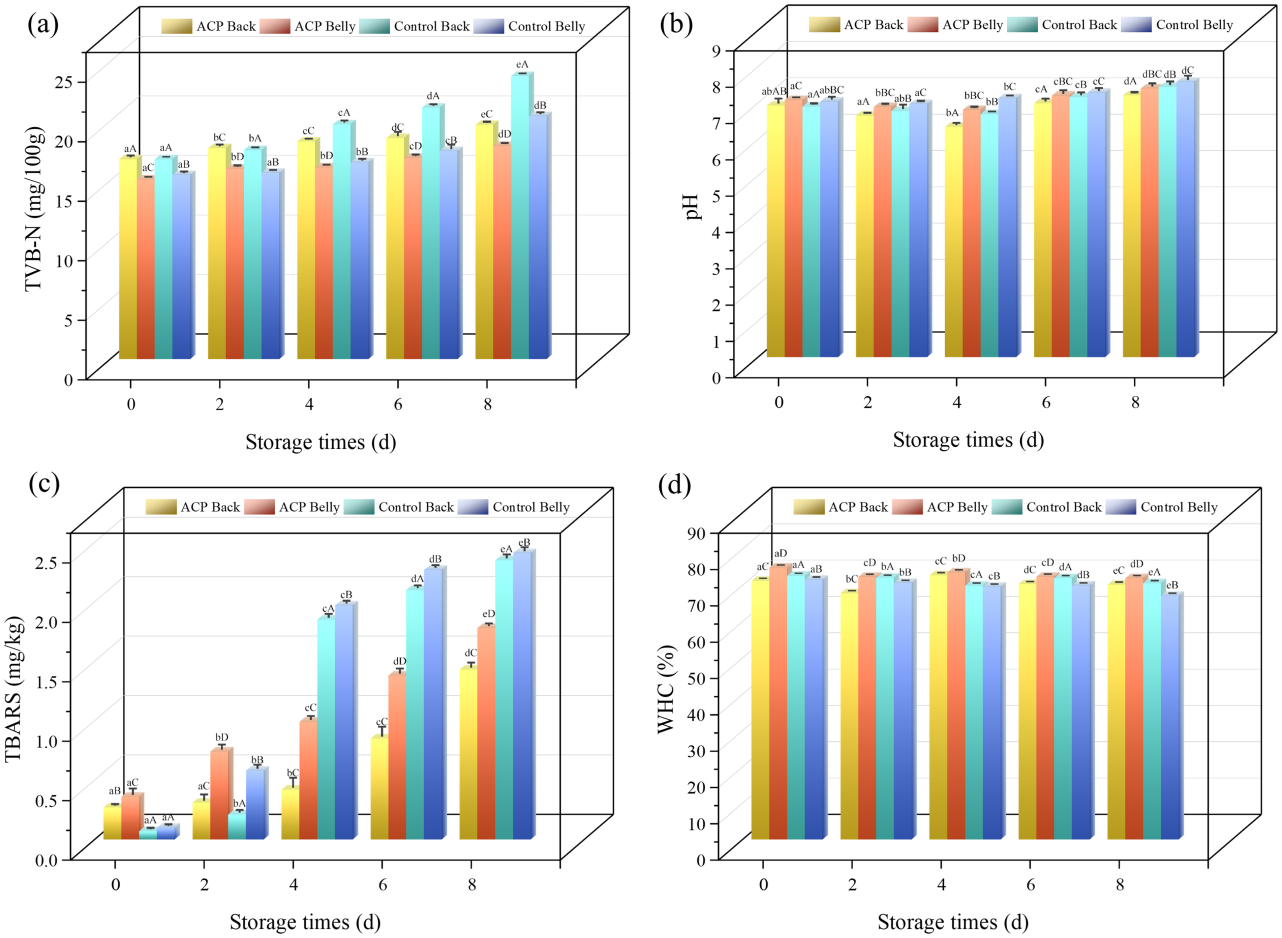
Changes in **(a)** TVB-N, **(b)** pH, **(c)** TBARS, **(d)** water holding capacity of large yellow croaker samples during refrigeration in different treatments. ^a–e^ Represents within-group differences. ^A–D^ Represents between-group differences.

TVB-N value is a key indicator for assessing fish freshness: <30 mg/100 g meets the edible standard, while <13 mg/100 g indicates premium-grade freshness (19). In this study, all samples were not deteriorated during storage (TVB-N values below 30 mg/100 g). The results showed that initial TVB-N values of each group ranged from 15.10 to 16.79 mg/100 g. With prolonged storage duration, both the control group and the ACP group exhibited an increasing trend in TVB-N values. It was attributed to intensified protein breakdown by spoilage microorganisms and endogenous enzymatic activity (19). The 2019 study by Chen et al. indicated that microbial growth causes a rapid increase in TVB-N, and ACP treatment may be able to inhibit microbial growth (14). Methionine and tyrosine are important components of fish proteins and are essential for maintaining the nutritional value of fish meat (20). Xie et al. study found that the degradation of methionine and tyrosine leads to elevated TVB-N values in fish (21). As can be seen from the Figure 1a, the TVB-N value of the dorsal muscle in large yellow croaker is consistently higher than that of the abdominal muscle, both in the control and ACP-treated groups. This difference may result from the lower pH of the dorsal meat, which enhances acidic protease activity in weakly acidic conditions, accelerating the decomposition of methionine and tyrosine (22). ACP treatment effectively slowed the rate of TVB-N increase, and this effect was observed in both the dorsal and abdominal muscle. Specifically, in the dorsal muscle, the TVB-N value increased by 41.0% in the control group, while it only increased by 17.2% in the ACP-treated group, representing a reduction of 23.8%. In the abdominal muscle, the control group showed a 32.0% increase, whereas the treated group exhibited an 18.7% increase, corresponding to a reduction of 13.3%. Although the effect of ACP treatment was not significant during the early storage period, its inhibitory effect on TVB-N generation rate began to manifest from the fourth day onward, showing a statistically significant difference compared to the control group (*p* < 0.05).

In the present study, from Figure 1b, the pH values of large yellow croaker flesh exhibited an initial decline followed by gradual recovery during storage. Upon fish death, intracellular ATP undergoes degradation into ADP, AMP, and inosinic acid, releasing phosphate and hydrogen ions that decrease the pH of fish muscle tissue. Simultaneously, anaerobic glycolysis of muscle glycogen produces lactic acid, further contributing to acid accumulation. This process commences immediately postmortem and continues through the initial freezing and thawing phases, leading to a rapid pH decline (21). Several factors contribute to the pH elevation in fish during advanced storage: the accumulation of alkaline substances, enhanced microbial activity, the depletion of acidic compounds (e.g., lactic acid), the buffering effect of protein degradation products, and the influence of fat oxidation by-products. This phenomenon serves as a critical indicator of fish spoilage, often closely associated with the deterioration of fish quality and a decline in food safety (21, 23). In dorsal muscle tissue, the pH of treated groups ranged from 6.93 to 7.21, while control groups exhibited a range of 6.90–7.44. In abdominal muscle tissue, the pH of treated groups varied between 7.08 and 7.40, compared to 7.04–7.58 in control groups. The observed pH depression in ACP-treated fish tissue relative to controls under identical conditions demonstrates the antimicrobial efficacy of plasma-generated ROS and H₂O₂, generated during the treatment process on microbial metabolic activity. Consequently, these effects mitigated the pH elevation typically driven by spoilage-associated enzymatic reactions (7).

### 3.2 TBARS

TBARS (thiobarbituric acid reactive substances) measures lipid peroxidation by quantifying TBA-reactive secondary oxidation products. As showed in Figure 1c, ACP-treated fish specimens exhibited markedly elevated TBARS levels compared to controls during the initial phase, This phenomenon likely stems from ACP-generated reactive oxygen/nitrogen species (RONS) accelerating the formation and accumulation of secondary lipid oxidation products, and the same phenomenon was observed in the ACP-treated dry cured black carp (24). Although an initial increase in TBARS values has been observed, this phenomenon must be interpreted with caution. The TBARS assay primarily reflects secondary carbonyl compounds represented by malondialdehyde and may not fully capture early primary products of lipid oxidation (such as hydrogen peroxides). Consequently, while an initial rise in TBARS indicates the initiation of oxidative stress, the biological significance of its absolute value requires comprehensive assessment in conjunction with other oxidative stress markers. From the fourth day onwards, comparative analysis revealed substantially elevated TBARS levels in control fish relative to their ACP-treated counterpart, indicating that ACP can effectively delay secondary oxidation (14). TBARS values were consistently higher in abdominal part of fish than in dorsal muscle in both the control and ACP groups, which may be caused by the high fat content of abdominal fish. In summary, ACP treatment exhibits a dual effect on lipid oxidation: initially accelerating the formation of secondary lipid oxidation products, while subsequently effectively suppressing secondary oxidation. The dual effect of ACP observed in this study “initially promoting and subsequently inhibiting lipid oxidation” can be coherently explained by the dynamic changes in TBARS values and fatty acid composition. Reactive oxygen species (ROS) likely preferentially attack and oxidize the most reactive polyunsaturated fatty acids within lipids, thereby reshaping the composition of residual lipids. This renders them more resistant to subsequent oxidation, ultimately providing a more stable oxidative microenvironment for the overall lipids during prolonged storage.

### 3.3 WHC

Water holding capacity (WHC) is fundamentally correlated with the intrinsic moisture states of seafood matrices. Therefore, this critical rheological property serves not only as a key assessment criterion for quality characterization but also as a direct indicator of its economic benefits in the market circulation (25). As shown in Figure 1d, WHC measurements indicated a declining trend in water-holding capacity of large yellow croaker muscle during the experimental period. Regional differences in WHC were evident, with abdominal muscle exhibiting accelerated deterioration rates compared to dorsal flesh in all test conditions. This differential may stem from the abdominal muscle’s higher fat content, where lipid oxidation generates reactive byproducts that compromise protein structure. These oxidative modifications impair protein-water interactions, ultimately exacerbating water-holding capacity loss (26).

Enhanced water holding capacity of dorsal muscle and abdominal muscle after ACP treatment on the fourth day of storage is consistent with the findings of Koddy et al. The mechanism is that ACP treatment promotes the unfolding of fish polysaccharide chains and proteins, exposing the internal hydrophilic groups, thus increasing the binding sites for water molecules in the fish and thus enhancing water holding capacity (27). At the same time, ACP treatment will trigger changes in the protein structure, forming a more dense and stable three-dimensional network structure, water molecules are more effectively wrapped in the network space formed by protein molecules, difficult to move freely and loss, thus improving the water holding capacity (28). The same increase in water holding capacity was observed in both parts of the control fish on the sixth day of storage, which we attributed to the increase in the length of the muscle segments and the space between the filaments during storage, which resulted in a larger space for water molecules, leading to an increase in the water holding capacity (29). However, as storage duration progressed, the protein in the fish will be overly denatured, as the storage cycle reached its final stage, the fish produces an unpleasant odor in the sensory, the structural network of the protein becomes dense, and the water is extruded, while the myofilaments undergo rupture and dissolution, and the water-holding capacity of the fish decreases.

### 3.4 Ca^2^^+^-ATPase

**Figure 2:**
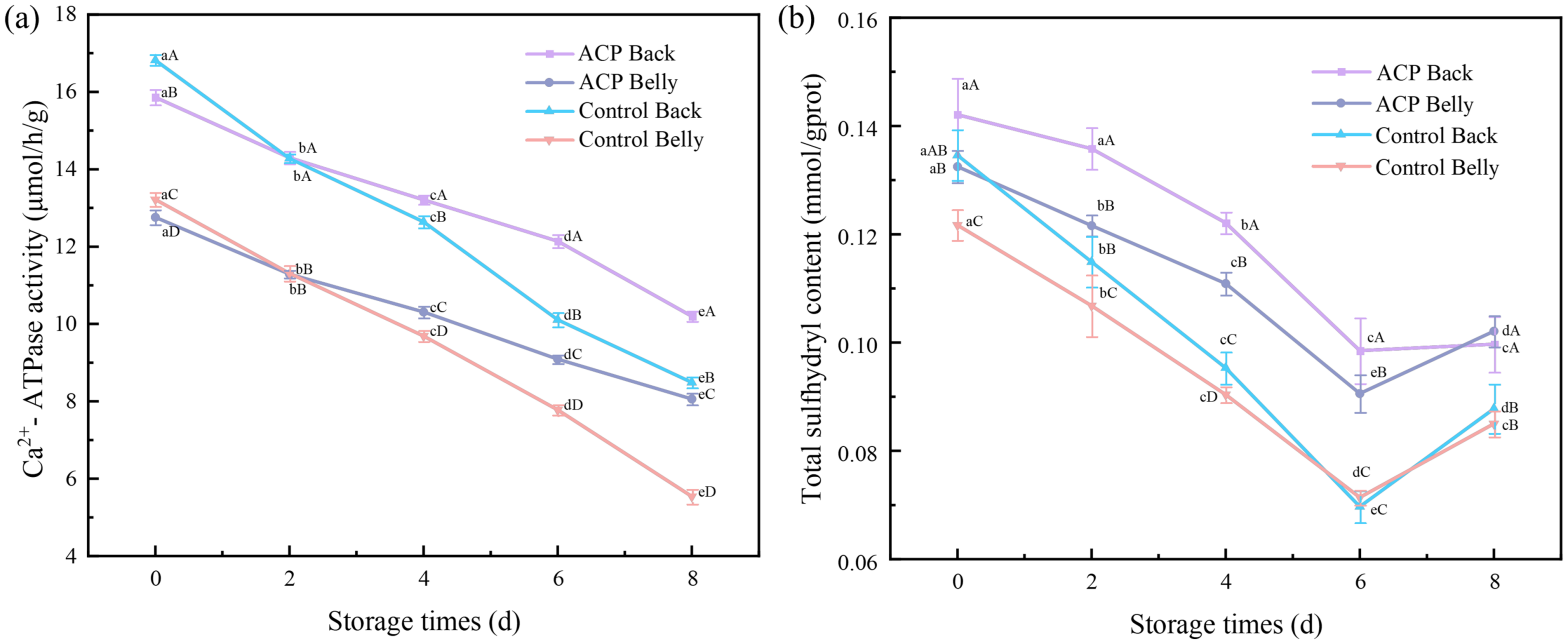
Changes in **(a)** Ca^2+^-ATPase activity **(b)** and total sulfhydryl content of large yellow croaker samples from different treatments during refrigeration.

The Ca^2+^-ATPase activity of myosin is commonly employed as a biochemical marker for monitoring myosin denaturation, a critical parameter that shows strong correlation with the textural quality of fish muscle (30). It was shown that sarcoplasmic reticulum Ca^2+^-ATPase activity showed a significant correlation with the structural stability of myofibrillar protein (MP), and that in general Ca^2+^-ATPase and total sulfhydryl groups often follow the same trends (31). As illustrated in Figure 2a, relative to abdominal muscles, dorsal muscle demonstrated enhanced Ca^2+^-ATPase activity in every experimental group. ACP processing yielded dual benefits: elevated Ca^2+^-ATPase activity (*p* < 0.05) and attenuated activity loss, indicating effective stabilization.

### 3.5 Total sulfhydryl group

The content of sulfhydryl groups (-SH) is a core indicator to characterize the degree of protein oxidation during fish storage. As the active functional groups of cysteine residues in myofibrillar proteins (MP), sulfhydryl groups exist in the form of hidden hydrophobic sulfhydryl groups within the molecule and surface-exposed reactive sulfhydryl groups, and their dynamic changes directly reflect the degree of cross-linking and polymerization, conformational rearrangement and irreversible denaturation of protein molecules (32). As illustrated in Figure 2b, the total sulfhydryl content in large yellow croaker flesh exhibited an initial decline, followed by a subsequent increase, and the decrease in sulfhydryl content indicated an increase in the oxidation of proteins in the fish flesh (11), exhibiting markedly elevated total sulfhydryl levels versus untreated groups (*p* < 0.05), denoting ACP’s protective effect on sulfhydryls, but the differences in total sulfhydryl content of the two different parts of the fish, namely, the abdomen and the dorsum, were not significant. Maintaining the protein conformational stability through non-thermodynamic effects, and decreasing the degree of exposure of the surface hydrophobic groups, thus effectively inhibiting the oxidative denaturation reaction of proteins triggered by environmental factors during the storage process. A notable resurgence in total sulfhydryl content emerged by day 8 of storage, and it was hypothesized that the mechanism might be related to the production of extracellular enzymes with sulfhydryl reduction function (e.g., thioredoxin reductase, glutathione reductase, etc.) and small-molecule reducing metabolites (e.g., hydrogen sulfide, glutathione, etc.) by specific microbial colonies through the mechanism of adaptive regulation, and that these biologically active substances could effectively reverse the oxidative modification of protein sulfhydryl groups, thus restore the homeostatic level of sulfhydryl compounds.

### 3.6 Sensory analysis

**Figure 3:**
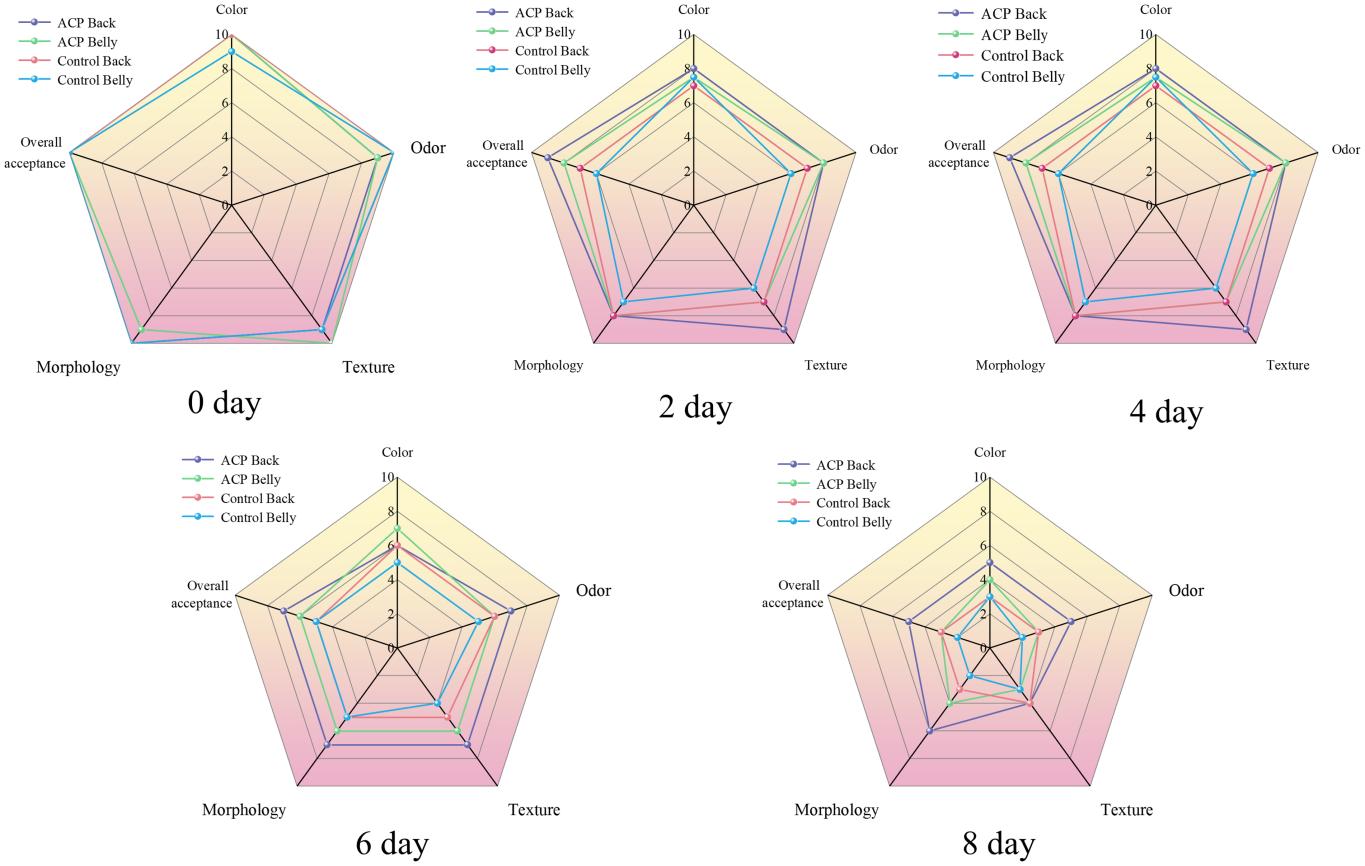
Changes in sensory scores of large yellow croaker samples from different treatments during refrigeration.

The initial evaluation results of the large yellow croaker samples by the sensory evaluation panel demonstrated consistency across five dimensions: color, odor, texture, appearance, and overall acceptability. As illustrated in Figure 3, the overall sensory evaluation scores between the ACP-treated group and the control group exhibited minimal differences on 0 d of storage, demonstrating that short-term ACP application had no discernible influence on sensory profiles. During the subsequent storage period, the sensory scores for both groups exhibited a continuous downward trend, with the quality deterioration of the control group becoming significantly pronounced from the fourth day onward. This deterioration was characterized by a gradual change in meat color from the initial light pink to grayish-white, accompanied by the emergence of undesirable odors. By the sixth day of storage, the control samples displayed evident greenish coloration, disintegration of the muscle fiber structure, and the release of characteristic odors associated with typical protein spoilage. Throughout the storage period, the sensory scores of the treated group consistently outperformed those of the control group, particularly in terms of odor deterioration inhibition. This can be attributed to the effective reduction of volatile alkaline nitrogenous substances production through the dual mechanism of inhibiting microorganism proliferation and blocking protein decomposition facilitated by ACP treatment (33).

### 3.7 Color and texture

Fish color reflects myoglobin redox status, lipid peroxidation, and microbial metabolite accumulation in muscle tissue, serving as a primary freshness indicator for consumers. In meat science, color stability is a critical quality attribute (34), that influences protein conformation and lipid oxidation (35), ultimately determining product marketability and commercial value.

**Figure 4:**
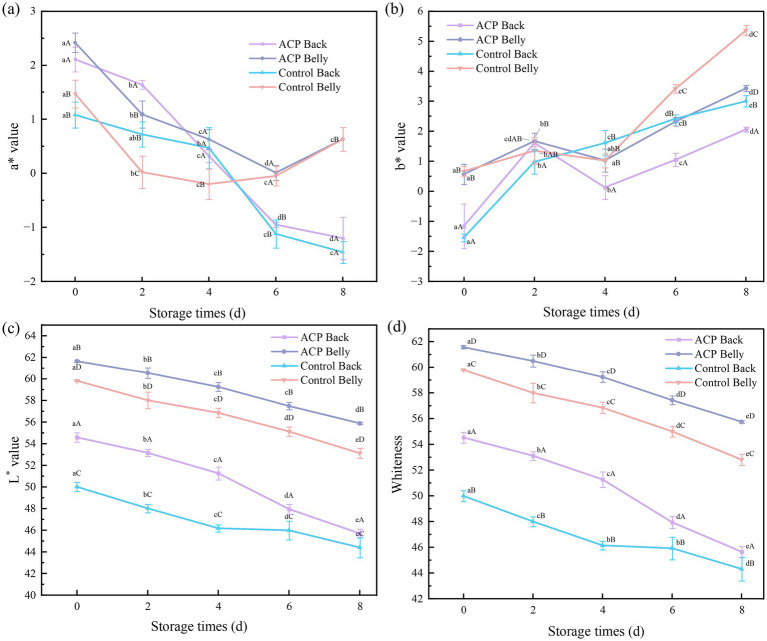
Changes in large yellow croaker samples with different treatments during refrigeration **(a)** a*, **(b)** b*, **(c)** L*, **(d)** whiteness.

From Figure 4a a* values of abdominal fish under various treatment conditions exhibited a trend of initially decreasing, followed by increasing. Dorsal a* values progressively declined during storage, unaffected by ACP treatment (*p* > 0.05). By day 8, ventral regions surpassed dorsal in a* values—potentially through hemoglobin/myoglobin oxidation (36), consistent with TBARS findings, abdominal fish exhibited more pronounced lipid peroxidation than dorsal counterparts at 8d. There is a close relationship between the significant fluctuations in a* values and the lipid peroxidation process, which has been extensively studied. The underlying mechanisms have been systematically elucidated through numerous empirical studies (37).

As illustrated in Figure 4b, the overall trend indicates an increase in b* of large yellow croaker, alongside a statistically significant elevation in b* values. This phenomenon may be attributed to the formation of oxidized heme proteins, such as high iron myoglobin, which can impart a greenish hue in Fe^3+^-porphyrin complexes due to d-d electron-leaping properties (37); at the same time, the oxidation of myoglobin mediated by hydrogen peroxide (H₂O₂) may promote the production of biliverdin derivatives through the Fenton mechanism, which in turn synergistically enhances the yellow coloration, a phenomenon that was similarly demonstrated in the study of Dinesh et al. (38). Notably, the b* values of the other three groups, excluding the control dorsal fish, decreased on the fourth day of storage. During the initial storage phase, anaerobic fermentation of fish glycogen yields lactic acid, coupled with ATP decomposition to produce IMP and phosphate, which collectively lead to a significant reduction in muscle pH (39). This decline alters the protein structure, affecting pigment binding and resulting in decreased b* values. The subsequent recovery may be attributed to protein decomposition, producing nitrogenous compounds that raise pH, thereby facilitating the recovery of b* values. Although acute ACP treatment showed no immediate effect on b* values (0 d, *p* > 0.05), it effectively attenuated b* value increments during prolonged storage (8 d, *p* < 0.05 versus controls). A distinct spatial gradient was observed, with dorsal musculature consistently exhibiting reduced b* values compared to ventral counterparts.

As illustrated in Figures 4c,d the L* and Whiteness values in the color difference continued to decrease as the storage time became longer, and under the same treatment conditions, the abdominal flesh of large yellow croaker presented markedly elevated L* and whiteness indices relative to dorsal counterparts (*p* < 0.05), with treated samples maintaining superior luminosity and whiteness at both storage initiation (0 d) and conclusion (8 d) (*p* < 0.05), showing that ACP treatment was effective in protecting the color of the fish during the storage period.

As shown in Table 1, All large yellow croaker fish samples gradually became softer during storage and showed a decrease in hardness in the indexes, but the hardness of the abdominal tissues was always higher than that of the dorsal muscle, and the hardness and chewiness of ACP-treated fish were significantly higher than that of the control group, but the changes in Adhesiveness, cohesion, and resilience were not obvious.

**Table 1:** Changes in the texture of different parts of fish with different treatments during storage.

Parameters	Times (d)	ACP back	ACP belly	Control back	Control belly
Hardness	0	451.59 ± 25.64^aA^	614.68 ± 42.77^aC^	407.18 ± 51.80^aA^	538.49 ± 27.28^aC^
	2	426.32 ± 42.81^aA^	580.21 ± 6.65^aB^	398.96 ± 52.09^aA^	426.10 ± 17.21^aB^
	4	405.03 ± 11.62^aB^	507.23 ± 5.55^bC^	363.96 ± 33.53^abA^	407.12 ± 13.18^bC^
	6	357.97 ± 1.36^abA^	433.30 ± 9.29^cC^	306.09 ± 12.38^bcB^	370.77 ± 7.24^cC^
	8	270.65 ± 23.15^cA^	376.22 ± 22.55^dC^	248.6 ± 14.15^cA^	337.99 ± 11.00^dC^
Adhesiveness	0	16.70 ± 0.82^aA^	10.76 ± 0.62^aD^	21.41 ± 1.65^aB^	13.31 ± 0.26^aD^
	2	23.72 ± 23.72^bA^	12.65 ± 0.52^bD^	25.11 ± 0.50^bB^	13.9 ± 0.11^bD^
	4	25.08 ± 0.19^cA^	14.04 ± 0.10^cC^	27.07 ± 0.62^cB^	14.56 ± 0.28^cC^
	6	26.56 ± 1.15^dA^	15.01 ± 0.84^dC^	28.42 ± 0.47^cB^	15.66 ± 0.21^dC^
	8	30.20 ± 0.45^eA^	16.35 ± 0.19^eC^	30.45 ± 1.28^dB^	16.89 ± 0.18^eC^
Springiness	0	0.66 ± 0.02^aA^	0.69 ± 0.02^aA^	0.60 ± 0.03^aB^	0.66 ± 0.02^aA^
	2	0.62 ± 0.02^bA^	0.67 ± 0.03^abA^	0.62 ± 0.07^aA^	0.60 ± 0.02^abA^
	4	0.59 ± 0.01^bA^	0.63 ± 0.06^abA^	0.57 ± 0.01^abA^	0.61 ± 0.03^abA^
	6	0.50 ± 0.01^cA^	0.57 ± 0.01^bcBC^	0.53 ± 0.01^bAB^	0.5 ± 0.04^bcC^
	8	0.52 ± 0.02^cA^	0.59 ± 0.01^cB^	0.50 ± 0.01^bA^	0.50 ± 0.05^cAB^
Cohesiveness	0	0.50 ± 0.01^aA^	0.58 ± 0.02^aB^	0.48 ± 0.01^aA^	0.57 ± 0.04^aB^
	2	0.47 ± 0.01^bA^	0.56 ± 0.01^aD^	0.45 ± 0.01^bB^	0.53 ± 0.01^bB^
	4	0.45 ± 0.00^bcB^	0.55 ± 0.02^abC^	0.42 ± 0.01^cB^	0.54 ± 0.02^cB^
	6	0.40 ± 0.01^dA^	0.52 ± 0.02^cB^	0.43 ± 0.02^bcA^	0.52 ± 0.02^bcA^
	8	0.43 ± 0.02^cdA^	0.51 ± 0.01^bcC^	0.44 ± 0.02^bcA^	0.48 ± 0.02^bcA^
Chewiness	0	127.10 ± 21.52^aA^	220.60 ± 31.71^aB^	124.29 ± 16.58^aA^	211.99 ± 17.84^aB^
	2	106.85 ± 7.70^bA^	203.9 ± 31.47^abB^	102.31 ± 2.66^bA^	211.99 ± 17.84^bB^
	4	92.27 ± 2.60^bcA^	171.51 ± 12.90^bcB^	82.10 ± 8.98^cA^	154.36 ± 11.17^cB^
	6	90.87 ± 5.35^bcA^	159.4 ± 12.26^cD^	76.97 ± 5.17^cdB^	154.36 ± 11.17^dc^
	8	78.24 ± 5.8^cB^	138.32 ± 5.16^cC^	61.07 ± 2.75^dA^	86.43 ± 3.20^eB^
Resilience	0	1.41 ± 0.12^aA^	2.22 ± 0.02^aD^	1.17 ± 0.08^aB^	1.91 ± 0.04^aC^
	2	1.24 ± 0.05^bA^	2.04 ± 0.13^bD^	1.17 ± 0.08^bB^	1.75 ± 0.04^bC^
	4	1.03 ± 0.00^cA^	1.92 ± 0.05^bcC^	0.99 ± 0.01^cA^	1.78 ± 0.10^bB^
	6	0.90 ± 0.04^dA^	1.77 ± 0.13^cC^	0.98 ± 0.01^cA^	1.59 ± 0.06^Cb^
	8	0.84 ± 0.01^dA^	1.44 ± 0.03^dC^	0.85 ± 0.04^dA^	1.32 ± 0.09^dB^

^a–e^Represents within-group differences, ^A–D^ represents between-group differences.

### 3.8 Water migration

**Figure 5:**
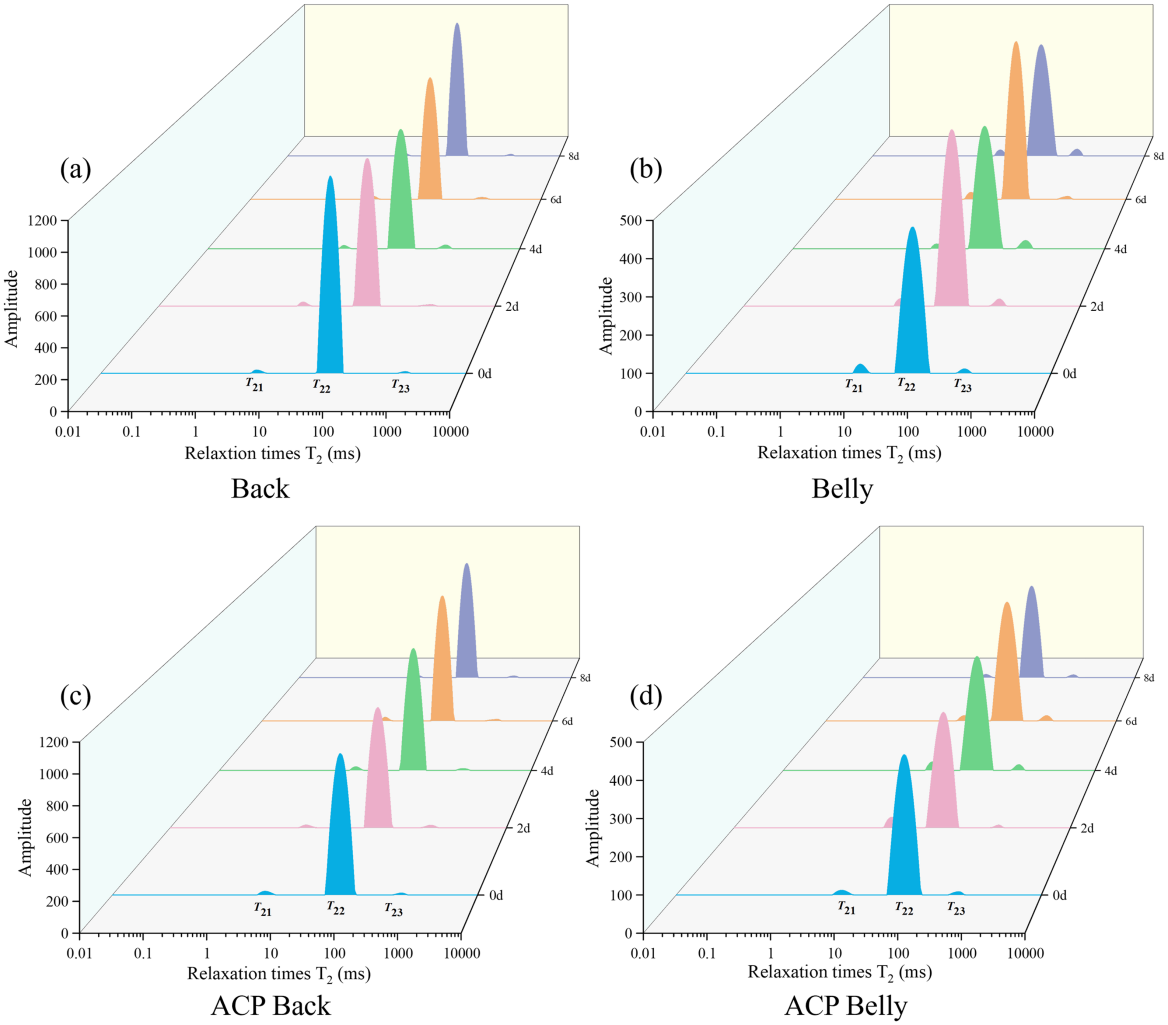
Changes in relaxation time of T_2_ curves for different parts of the large yellow croaker samples from different parts of the fish during refrigeration **(a)** back, **(b)** belly, and treated with the same ACP during cold storage **(c)** ACP back, **(d)** ACP belly.

Water migration characteristics of the dorsal and abdominal muscle tissues of the large yellow croakers were quantified using low-field nuclear magnetic resonance (NMR). The transverse relaxation signal (T_2_) was acquired and resolved by an inversion algorithm to obtain three-component water relaxation spectra with the following characteristic parameters: T_21_ (0.1-10 ms, corresponding to tightly bound water), T_22_ (10–100 ms, reflecting intra-myofibrillar bound water) and T_23_ (100–1,000 ms, characterizing the extracellular free water) (40). The integral values of each relaxation peak area accurately characterized the relative contents of water molecules in different fugitive states, which provided a quantitative basis for analyzing the water distribution characteristics of fish muscle tissues. The results obtained are shown in Figure 5 T_22_ levels in dorsal musculature consistently surpassed abdominal measurements during storage. Neither T_21_ nor T_23_ responded to ACP treatment, though it provided partial T_22_ stabilization versus untreated specimens.

As shown in Supplementary Table 1, the concentrations of pT_21_ and pT_22_ exhibited a decline with prolonged storage duration, with samples of T_22_ comprising 92.814–98.479% of the total water content. In contrast, the concentration of T_23_ showed a gradual increase across all samples. During extended storage, the muscle tissue of the fish became increasingly loose, resulting in decreased hardness and viscosity, which contributed to the loss of water in T_21_ and T_22_, as well as the conversion of bound water into free water (41). The ACP-treated dorsal muscle exhibited a significantly lower T23 proportion compared to the ventral muscle, indicating a higher free water loss rate in the dorsal portion. This finding is consistent with the previous conclusion that the water-holding capacity of treated dorsal muscle was inferior to that of ventral muscle. Relative to the control group, the treated group showed higher T23 proportions, demonstrating reduced free water loss. These results suggest that ACP treatment maintains muscle water-holding capacity by minimizing free water loss.

**Figure 6:**
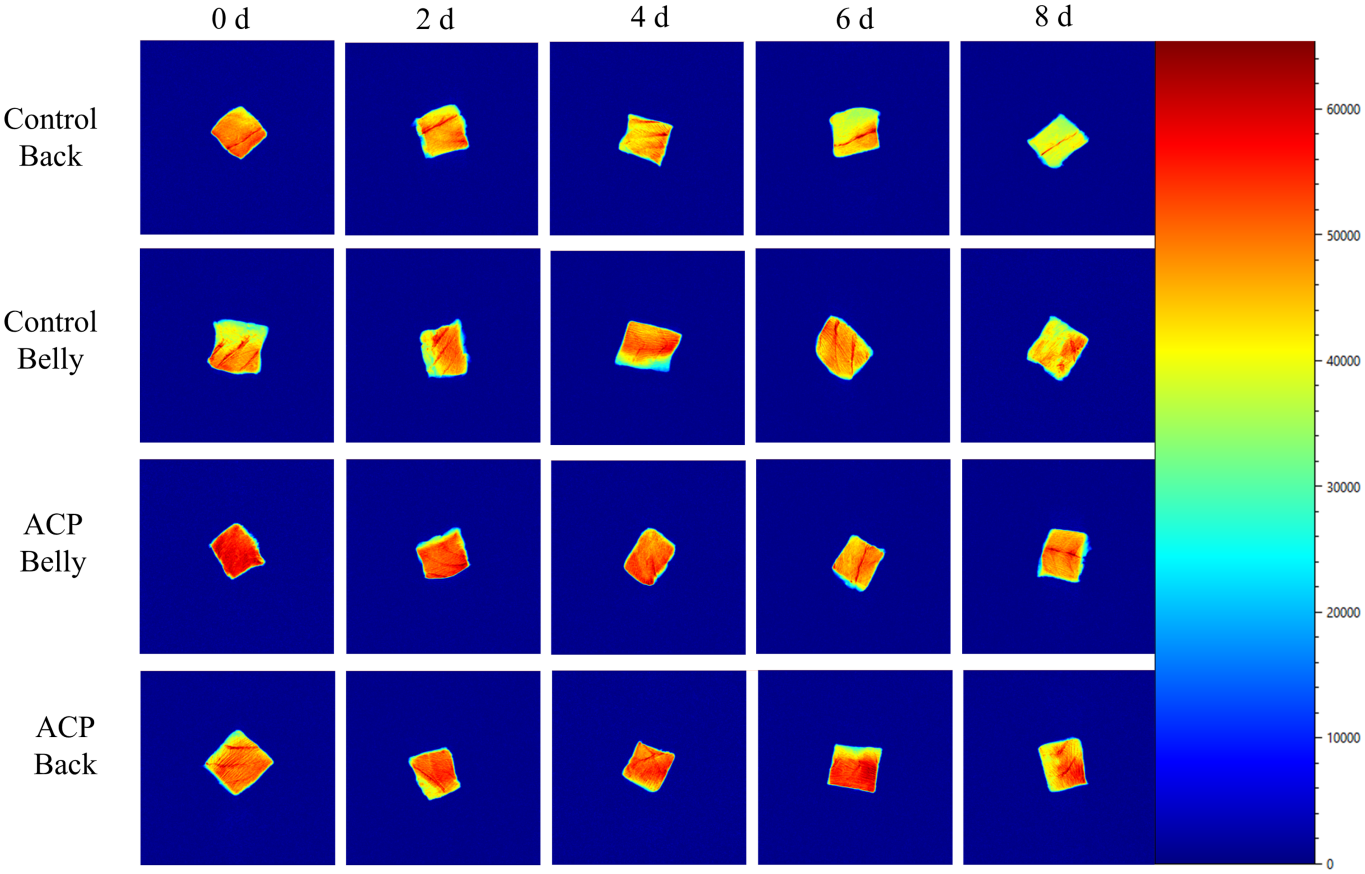
Changes in NMR images of large yellow croaker samples subjected to different treatments during refrigeration.

Magnetic Resonance Imaging (MRI) visualizes the spatial distribution of moisture in fish through transverse relaxation-weighted imaging, where red indicates regions of high hydrogen proton density and blue represents areas of low density (21). The results presented in Figure 6, demonstrate that as storage time increased, the red area of the fish meat significantly decreased across all groups. This finding suggests that substantial moisture migration and loss occurred, during prolonged storage, the ACP-treated fish muscle exhibited significantly higher a* and L* values compared to the control group (*p* < 0.05), demonstrating that ACP treatment effectively inhibited oxidative degradation of myofibrillar proteins and maintained the microstructural integrity of muscle tissue. These findings were consistent with the water distribution characteristics determined by low-field nuclear magnetic resonance (LF-NMR) and water-holding capacity (WHC) measurements, further validation of ACP treatment on the maintenance of fish quality in Large yellow croakers.

### 3.9 Analysis of fatty acid

**Figure 7:**
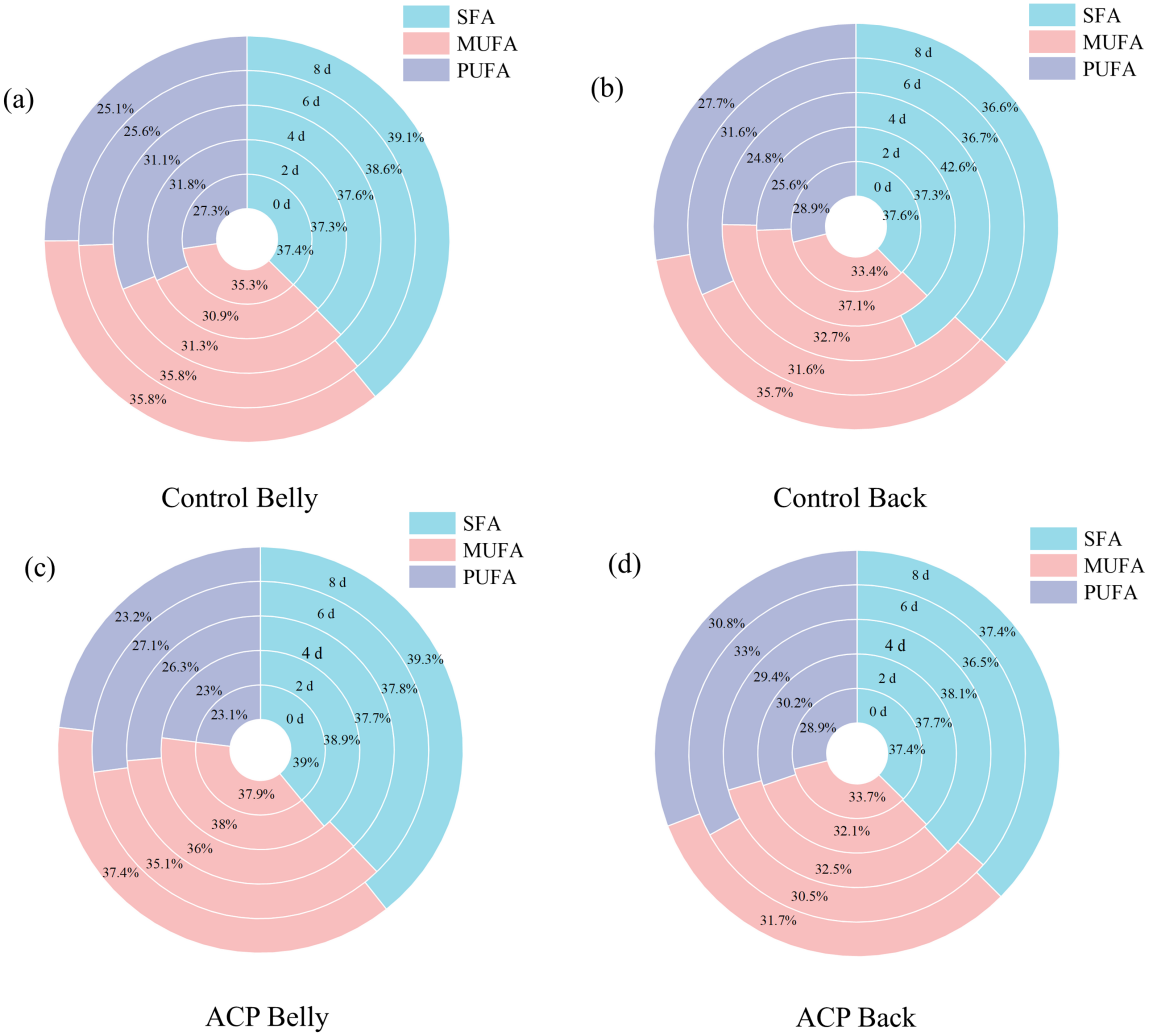
Changes in fatty acid composition in samples of large yellow croaker fish meat after different treatments during storage: **(a)** control abdominal muscle, **(b)** control dorsal muscle, **(c)** treatment abdominal muscle, **(d)** treatment dorsal muscle.

The present study aimed to investigate the effects of ACP treatment on the fatty acid composition of large yellow croaker muscle during cold storage. From Supplementary Table 2, a total of 27 fatty acids were detected in the flesh of the current batch of large yellow croaker. Among these, C14:0, C16:0, and C18:0 were identified as the predominant saturated fatty acids (SFA). In the abdominal flesh of the control fish, the levels of these three major fatty acids initially decreased before gradually rebounding. In contrast, the fluctuation of fatty acid content in the dorsal flesh was minimal, indicating a stabilization of dorsal fat metabolism. Of particular note, the ventral muscle of ACP-treated fish demonstrated substantially greater concentrations of these three fatty acids relative to control samples. This suggests that ACP treatment may inhibit lipid decomposition and oxidation during storage, thereby providing a protective effect on saturated fatty acids. C16:1 and C18:1n9c were the primary monounsaturated fatty acids (MUFA), and with extended storage time, the content of C16:1 in the abdominal flesh of ACP-treated fish on day 8 showed a 52% elevation relative to control values. ACP treatment also appeared to delay the decreasing trend of C18:1n9c in the abdominal flesh, although it had a limited effect on the dorsal region. C18:2n6c and C22:6n3 were identified as the main polyunsaturated fatty acids (PUFA); ACP treatment significantly enhanced the accumulation of C18:2n6c in the abdominal region, particularly at days 4 and 8, while inhibiting the content of this fatty acid in the dorsal flesh. Furthermore, the abdominal flesh exhibited greater sensitivity to ACP treatment compared to the dorsal flesh. As indicated in Figure 7, In large yellow croaker abdominal muscle, ACP intervention substantially lowered PUFA content compared to untreated samples, while elevating SFA and MUFA proportions at day 0—evidence of PUFA transformation.

### 3.10 Characterization of volatile compounds in the flesh of the large yellow croaker by the GC–MS method

**Figure 8:**
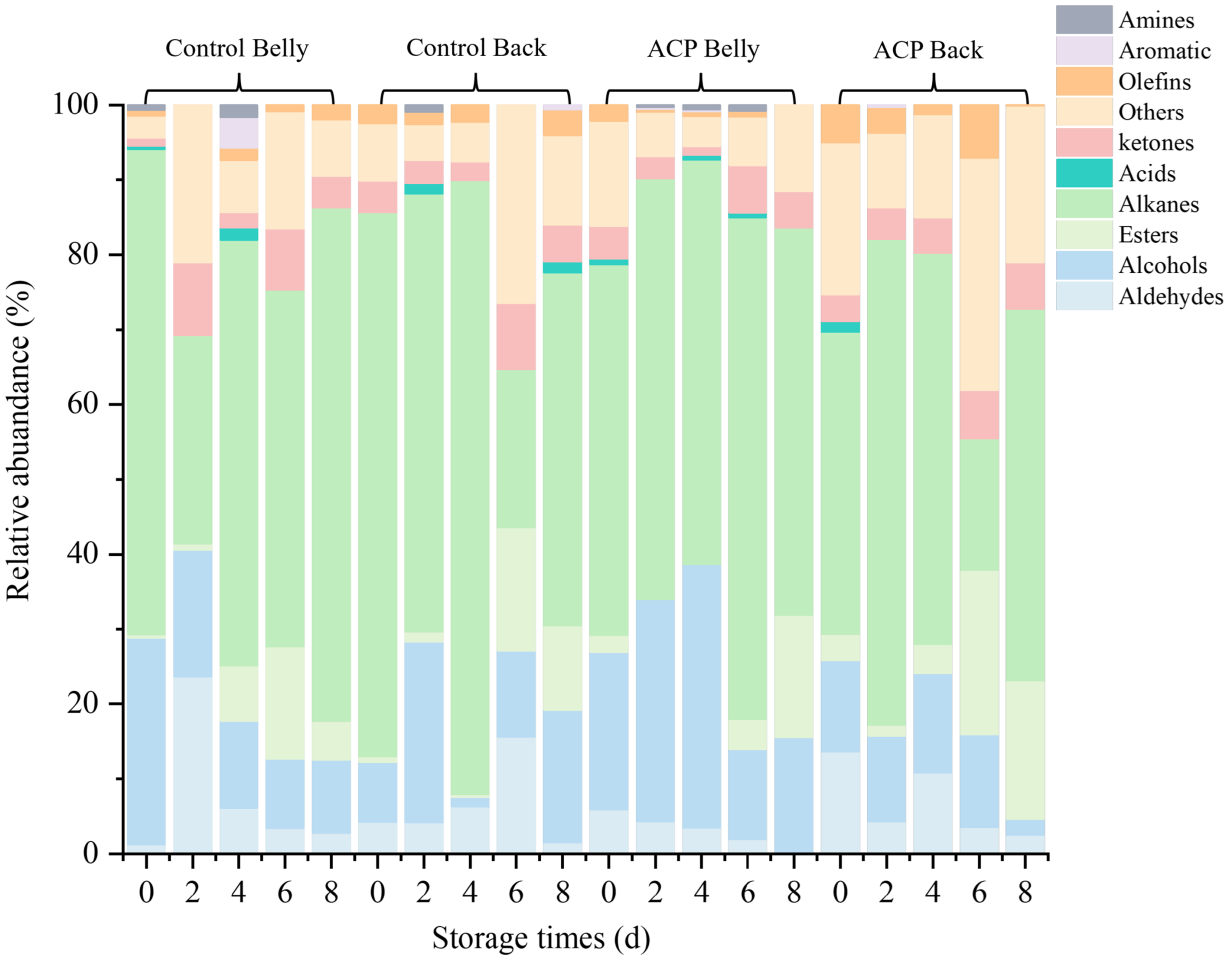
Volatile compounds stacking plots of fish samples from different treatments during the storage period.

A systematic analysis of the volatile flavor compositions of muscle tissues from different parts of the large yellow croaker during storage was carried out by gas chromatography–mass spectrometry (GC–MS). A total of 60 volatile flavor compounds were identified across the 20 samples in this study, including 30 alkanes, 7 alcohols, 4 aldehydes, 4 esters, 4 ketones, 1 aromatic hydrocarbon, 1 amine, 1 acid, and 4 other compounds. As illustrated in Figure 8, At the 0 d, it can be observed that the compositional differences of volatile components between the treatment group and the control group were not significant, the types of volatile compounds in the fish changed significantly as storage duration increased. Notably, alkanes and alcohols predominated in all tested samples, exhibiting significantly higher relative contents compared to other classes of volatiles.

**Figure 9:**
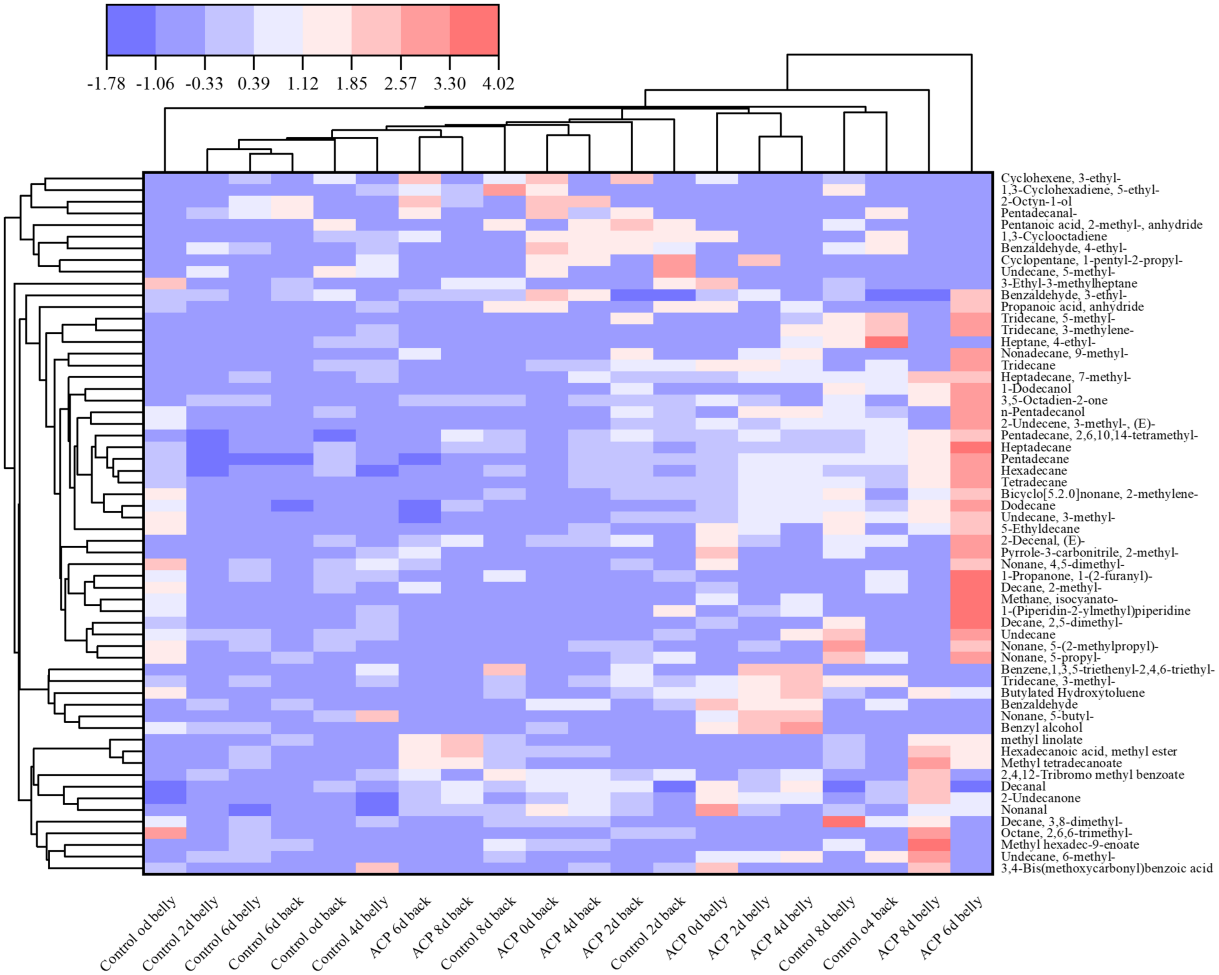
Thermogram for visualization of volatile compounds in fish samples with different treatments during storage period.

Furthermore, the volatile heat map Figure 9, alkanes, identified as the most significant volatile compounds in this study, constitute an essential part of the volatile organic components of aquatic products (42). Existing research has demonstrated that these compounds serve not only as precursor substances for characteristic flavor compounds in meat but also as important intermediates in the formation of key flavor heterocyclic compounds through pathways such as the Maillard reaction (43).

Esters are primarily formed through the esterification process involving the condensation of short-chain saturated fatty acids and saturated fatty alcohols. Most esters exhibit fruity and floral aromas, these changes mediate flavor modulation through pronounced attenuation of short-chain fatty acids’ pungent rancidity and amines’ characteristic bitterness (44). Alcohols represent the second most abundant group of volatile compounds found in the flesh of yellow croaker, primarily resulting from the degradation of unsaturated fatty acids. Aldehydes are key products in the oxidative degradation of lipids (45). In this study, benzaldehyde is noted for its characteristic bitter almond-cherry aroma complemented by sweet nutty and fruity undertones (46). Benzaldehyde, 3-ethyl-, resembles benzaldehyde but possesses stronger caramel, woody, and slightly smoky notes. Benzaldehyde, 4-ethyl-, is characterized by its vanilla and honey sweetness, while 2-Decenal, (E)-, is recognized for its strong fat-oxidizing odor, which has a very low threshold and contributes to a pronounced off-flavor.

A total of four ketones were identified in the flesh of yellow croaker, specifically 3,5-Octadien-2-one and 2-Undecanone, both of which imparted a floral aroma (43). Additionally, four olefins were detected, typically associated with sweet and aromatic scents (47). Notably, the thresholds for three of these olefins, excluding 3-Ethylcyclohexene, were relatively high, indicating that they contributed minimally to the fish’s overall flavor profile. Furthermore, at low concentrations, one of the substances exhibited a mushroom-like freshness.

### 3.11 PCA analysis

**Figure 10:**
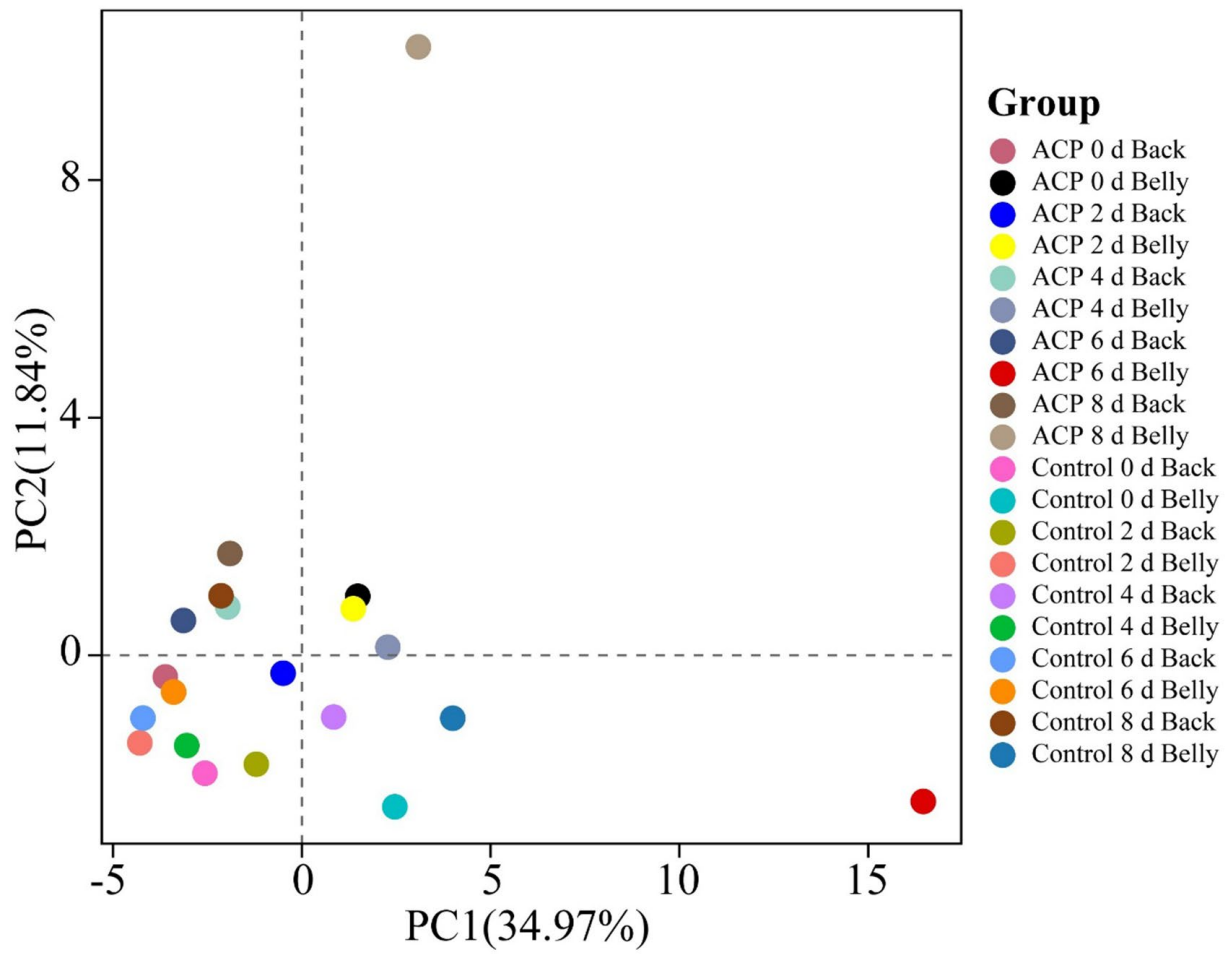
PCA analysis of volatile compounds.

Based on the volatile compounds detected in the 20 samples, the quantitative results of the classified compounds were subjected to PCA analysis, which was used to react to the differences between the samples, such as PC1 (spindle 1) and PC2 (spindle 2), etc. According to the results of Figure 10, the samples were roughly divided into three relatively independent regions, and when the samples showed an overlap or neighboring status in their spatial distributions, it indicated that the volatile odor components had a high degree of high similarity; conversely, an increase in the spatial distance between samples is positively correlated with the variability of their odor characteristics (43). Notably, the 6d and 8d samples of the ACP-treated abdomen exhibited significant differences from the other samples, with a clear distinction between these two. Although all samples shifted to the right along the PC1 axis over time, at the same storage time points, the sample points of the ACP-treated group were generally positioned further to the left on the PC1 axis compared to those of the control group. This indicates that, within the multivariate space, ACP treatment helped the samples retain metabolic characteristics closer to those of fresh samples, visually demonstrating its delaying effect on biochemical changes associated with spoilage. The spatial separation between the treated and control groups at identical time points provides holistic evidence for the preservative efficacy of ACP.

## 4 Conclusion

This study investigated the effects of ACP treatment on the quality of different fish muscle sections in large yellow croaker during 4 °C storage through multidimensional indicator analysis. The results of this study demonstrate that atmospheric cold plasma treatment (ACP, 40 kV, 90 s) significantly inhibited the accumulation of TVB-N and TBARS (*p* < 0.05) in fish samples while effectively mitigating the decline in water-holding capacity. Compared with the control group, the total sulfhydryl content in dorsal and ventral muscles increased significantly by 20 and 45%, respectively, and Ca^2+^-ATPase activity was also significantly enhanced by 14 and 20% (*p* < 0.05) after treatment. Furthermore, ACP treatment significantly maintained the color parameters (L, a, b*) and textural properties (hardness, elasticity, etc.) of fish meat (*p* < 0.05). Notably, although ACP treatment initially accelerated fat oxidation (particularly significant in abdominal fish meat), it ultimately proved effective in inhibiting secondary fat oxidation over extended storage periods. A total of 60 volatile compounds were identified in the fish samples using GC–MS, encompassing eight major substance groups, including alkanes, alcohols, and aldehydes. These findings collectively indicate that ACP treatment effectively delays quality deterioration in fish during refrigerated storage through multiple synergistic mechanisms. This study demonstrates the potential of ACP treatment in preserving the quality of large yellow croaker, yet several limitations should be noted. For instance, the storage period was limited to 0–8 days, and the effects of additional ACP treatment parameters were not explored. Additionally, the analysis focused on the abdominal muscle and dorsal muscle based on market consumption habits. Future research should extend the storage observation period, systematically optimize ACP parameters, and include whole-fish assessments to further validate and generalize these findings.

## Data Availability

The original contributions presented in the study are included in the article/Supplementary material, further inquiries can be directed to the corresponding author.

## References

[1] Mu H Li J Pan X Liu J Chen J Pan Y . Alterations in fatty acid composition and volatile compounds in muscle of large yellow croaker *Larimichthys Crocea* fed different dietary lipid sources. Aquac Rep. (2021) 20:100688. doi: 10.1016/j.aqrep.2021.100688

[2] Zhao J Lv W Wang J Li J Liu X Zhu J. Effects of tea polyphenols on the post-mortem integrity of large yellow croaker (*Pseudosciaena Crocea*) fillet proteins. Food Chem. (2013) 141:2666–74. doi: 10.1016/j.foodchem.2013.04.126, 23871009

[3] Laroussi M. Plasma-based Sterilization. In Proceedings of XXVI International Conference on Phenomena in Ionized Gases, Greifswald, Deutsche Physikalische Gesellschaft (2003).

[4] Chaomuang N Singphithak P Laguerre O Suwapanich R. Temperature control in a horticultural produce supply chain in Thailand and its influence on product quality. Food Control. (2022) 133:108585. doi: 10.1016/j.foodcont.2021.108585

[5] Rathod NB Ranveer RC Bhagwat PK Ozogul F Benjakul S Pillai S . Cold plasma for the preservation of aquatic food products: an overview. Compr Rev Food Sci Food Saf. (2021) 20:4407–25. doi: 10.1111/1541-4337.12815, 34355478

[6] Baier M Görgen M Ehlbeck J Knorr D Herppich WB Schlüter O. Non-thermal atmospheric pressure plasma: screening for gentle process conditions and antibacterial efficiency on perishable fresh produce. Innov Food Sci Emerg Technol. (2014) 22:147–57. doi: 10.1016/j.ifset.2014.01.011

[7] Muranyi P Wunderlich J Heise M. Sterilization efficiency of a cascaded dielectric barrier discharge. J Appl Microbiol. (2007) 103:1535–44. doi: 10.1111/j.1365-2672.2007.03385.x, 17953564

[8] Wang J Fu T Sang X Liu Y. Effects of high voltage atmospheric cold plasma treatment on microbial diversity of Tilapia (*Oreochromis Mossambicus*) fillets treated during refrigeration. Int J Food Microbiol. (2022) 375:109738. doi: 10.1016/j.ijfoodmicro.2022.109738 35635991

[9] Hu J Chen J Zheng Y Huang J Xie K Li Y . Effect of atmospheric cold plasma treatment modes on the quality of red shrimp (*Solenocera Crassicornis*) during cold chain storage. LWT. (2023) 190:115543. doi: 10.1016/j.lwt.2023.115543

[10] Wu Y Wu Q Lin H Pang J Zhou X Zhang B. Effects of cold atmospheric plasma pre-treatment on maintaining the quality of ready-to-eat drunken red shrimp (*Solenocera Crassicornis*) stored at chilled conditions. Food Chem X. (2023) 20:100934. doi: 10.1016/j.fochx.2023.100934 38144752 PMC10740073

[11] Cheng H Mei J Xie J. Stability of large yellow croaker (*Pseudosciaena Crocea*) as affected by temperature abuse during frozen storage: quality attributes, myofibril characteristics, and microstructure. Cryobiology. (2024) 117:105157. doi: 10.1016/j.cryobiol.2024.105157 39477053

[12] Bian C Cheng H Yu H Mei J Xie J. Effect of multi-frequency ultrasound assisted thawing on the quality of large yellow croaker (*Larimichthys Crocea*). Ultrason Sonochem. (2022) 82:105907. doi: 10.1016/j.ultsonch.2021.105907 34998136 PMC8799743

[13] Zhao Y Lan W Shen J Xu Z Xie J. Combining ozone and slurry ice treatment to prolong the shelf-life and quality of large yellow croaker (*Pseudosciaena crocea*). Lwt. (2022) 154:112615. doi: 10.1016/j.lwt.2021.112615

[14] Chen J Wang S-Z Chen J-Y Chen D-Z Deng S-G Xu B. Effect of cold plasma on maintaining the quality of chub mackerel (*Scomber japonicus*): biochemical and sensory attributes. J Sci Food Agric. (2019) 99:39–46. doi: 10.1002/jsfa.9138 29786860

[15] Chu Y Cheng H Yu H Mei J Xie J. Quality enhancement of large yellow croaker (*Pseudosciaena crocea*) during frozen (−18 °c) storage by spiral freezing. CyTA J Food. (2021) 19:710–20. doi: 10.1080/19476337.2021.1960895

[16] Li H Wang Y Zhang J Li X Wang J Yi S . Prediction of the freshness of horse mackerel (*Trachurus japonicus*) using E-nose, E-tongue, and colorimeter based on biochemical indexes analyzed during frozen storage of whole fish. Food Chem. (2023) 402:134325. doi: 10.1016/j.foodchem.2022.134325 36174352

[17] Li B Wang X Gao X Mei J Xie J. Effect of active coatings containing Lippa citriodora Kunth. Essential oil on bacterial diversity and myofibrillar proteins degradation in refrigerated large yellow croaker. Polymers. (2021) 13:1787. doi: 10.3390/polym13111787 34071698 PMC8198210

[18] Zhang J Wu F Kasato Y Deng S Brennan C Benjakul S . Effect of atmospheric cold plasma treatment on the flavor of high-fat aquatic products: a case study of golden pomfret (*Trachinotus ovatus*, family Bramidae) oil using Gc-Ms, Gc-Ims, and an E-nose. Lwt. (2024) 210:116843. doi: 10.1016/j.lwt.2024.116843

[19] Zong L Gao H Chen C Xie J. Effects of starch/polyvinyl alcohol active film containing cinnamaldehyde on the quality of large yellow croaker (*Pseudosciaena crocea*) proteins during frozen storage. Food Chem. (2022) 389:133065. doi: 10.1016/j.foodchem.2022.133065 35489262

[20] Lu S. Effects of bactericides and modified atmosphere packaging on shelf-life of Chinese shrimp (*Fenneropenaeus chinensis*). LWT Food Sci Technol. (2009) 42:286–91. doi: 10.1016/j.lwt.2008.03.004

[21] Ma X Mei J Xie J. Effects of multi-frequency ultrasound on the freezing rates, quality properties and structural characteristics of cultured large yellow croaker (*Larimichthys crocea*). Ultrason Sonochem. (2021) 76:105657. doi: 10.1016/j.ultsonch.2021.105657 34229120 PMC8261011

[22] Abdilbar Usman SM Jermen Mamo. Production, optimization and characterization of an acid protease from filamentous fungi by solid-state fermentation. Res Square. (2020). 1–29. doi: 10.21203/rs.3.rs-94943/v1 PMC8102121 34007282

[23] Bu Y Han M Tan G Zhu W Li X Li J. Changes in quality characteristics of southern Bluefin tuna (*Thunnus maccoyii*) during refrigerated storage and their correlation with color stability. LWT. (2022) 154:112715. doi: 10.1016/j.lwt.2021.112715

[24] Ke Z Bai Y Chu Y Gu S Xiang X Ding Y . Cold plasma treated air improves the characteristic flavor of dry-cured black carp through facilitating lipid oxidation. Food Chem. (2022) 377:131932. doi: 10.1016/j.foodchem.2021.131932 34999450

[25] Szmanko T Lesiow T Gorecka J. The water-holding capacity of meat: a reference analytical method. Food Chem. (2021) 357:129727. doi: 10.1016/j.foodchem.2021.129727 33964628

[26] Alakali JS Mzer MT. Quality evaluation of beef patties formulated with Bambara groundnut (*Vigna subterranea* L.) seed flour. Meat Sci. (2010) 85:215–23. doi: 10.1016/j.meatsci.2009.12.027, 20374888

[27] Koddy JK Miao W Hatab S Tang L Xu H Nyaisaba BM . Understanding the role of atmospheric cold plasma (Acp) in maintaining the quality of hairtail (*Trichiurus lepturus*). Food Chem. (2021) 343:128418. doi: 10.1016/j.foodchem.2020.128418, 33160769

[28] Chanarat S Benjakul S. Impact of microbial transglutaminase on gelling properties of Indian mackerel fish protein isolates. Food Chem. (2013) 136:929–37. doi: 10.1016/j.foodchem.2012.09.021, 23122146

[29] Zeng Z Li C Ertbjerg P. Relationship between proteolysis and water-holding of myofibrils. Meat Sci. (2017) 131:48–55. doi: 10.1016/j.meatsci.2017.04.232 28463752

[30] Liang J Wang Z Zhou L Niu Y Yuan C Tian Y. Ultrastructural and biochemical changes of sarcoplasmic reticulum in spotted mackerel (*Scomber australasicus* Cuvier, 1832) muscle during cold storage at 5 °C. Int J Food Sci Technol. (2023) 58:3810–8. doi: 10.1111/ijfs.16482

[31] Shi L Yang T Xiong G Li X Wang X Ding A . Influence of frozen storage temperature on the microstructures and physicochemical properties of pre-frozen perch (*Micropterus salmoides*). LWT. (2018) 92:471–6. doi: 10.1016/j.lwt.2018.02.063

[32] Pei J Mei J Wu G Yu H Xie J. Gum tragacanth-sodium alginate active coatings containing epigallocatechin gallate reduce hydrogen peroxide content and inhibit lipid and protein oxidations of large yellow croaker (*Larimichthys crocea*) during superchilling storage. Food Chem. (2022) 397:133792. doi: 10.1016/j.foodchem.2022.133792 35917785

[33] Shiekh KA Benjakul S. Melanosis and quality changes during refrigerated storage of Pacific white shrimp treated with Chamuang (Garcinia cowa Roxb.) leaf extract with the aid of pulsed electric field. Food Chem. (2020) 309:125516. doi: 10.1016/j.foodchem.2019.125516 31708342

[34] Kono S Kon M Araki T Sagara Y. Effects of relationships among freezing rate, ice crystal size and color on surface color of frozen salmon fillet. J Food Eng. (2017) 214:158–65. doi: 10.1016/j.jfoodeng.2017.06.023

[35] Mancini RA Hunt MC. Current research in meat color. Meat Sci. (2005) 71:100–21. doi: 10.1016/j.meatsci.2005.03.003, 22064056

[36] Bekhit AE-DA Geesink GH Ilian MA Morton JD Sedcole R Bickerstaffe R. Particulate Metmyoglobin reducing activity and its relationship with meat color. J Agric Food Chem. (2003) 51:6026–35. doi: 10.1021/jf030093e 13129312

[37] Albertos I Martin-Diana AB Cullen PJ Tiwari BK Ojha KS Bourke P . Shelf-life extension of herring (*Clupea harengus*) using in-package atmospheric plasma technology. Innov Food Sci Emerg Technol. (2019) 53:85–91. doi: 10.1016/j.ifset.2017.09.010

[38] Jayasena DD Kim HJ In Yong H Park S Kim K Choe W . Flexible thin-layer dielectric barrier discharge plasma treatment of pork butt and beef loin: effects on pathogen inactivation and meat-quality attributes. Food Microbiol. (2015) 46:51–7. doi: 10.1016/j.fm.2014.07.009 25475266

[39] Walayat N Tang W Wang X Yi M Guo L Ding Y . Quality evaluation of frozen and chilled fish: a review. eFood. (2023) 4. doi: 10.1002/efd2.67

[40] Qian S Li X Wang H Mehmood W Zhong M Zhang C . Effects of low voltage electrostatic field thawing on the changes in physicochemical properties of myofibrillar proteins of bovine longissimus dorsi muscle. J Food Eng. (2019) 261:140–9. doi: 10.1016/j.jfoodeng.2019.06.013

[41] Wang X-Y Xie J. Study on the volatile organic compounds and its correlation with water dynamics of bigeye tuna (*Thunnus obesus*) during cold storage. Molecules. (2019) 24:3119. doi: 10.3390/molecules24173119 31466228 PMC6749214

[42] Li Y Yuan L Liu H Liu H Zhou Y Li M . Analysis of the changes of volatile flavor compounds in a traditional Chinese shrimp paste during fermentation based on electronic nose, SPME-GC-MS and HS-GC-IMS. Food Sci Human Wellness. (2023) 12:173–82. doi: 10.1016/j.fshw.2022.07.035

[43] Cheng H Mei J Xie J. Analysis of key volatile compounds and quality properties of tilapia (*Oreochromis mossambicus*) fillets during cold storage: based on thermal desorption coupled with gas chromatography-mass spectrometry (TD-GC-MS). LWT. (2023) 184:115051. doi: 10.1016/j.lwt.2023.115051

[44] Pinho O., CP, Ferreira I. M. P. L. V. O. Solid-phase microextraction of volatile compounds in “Terrincho” ewe cheese. J Chromatogr A (2003) 1011:1–9. doi: 10.1016/s0021-9673(03)01066-5. 14518757

[45] Xu X Lu S Li X Bai F Wang J Zhou X . Effects of microbial diversity and phospholipids on flavor profile of caviar from hybrid sturgeon (*Huso dauricus × acipenser schrencki*). Food Chem. (2022) 377:131969. doi: 10.1016/j.foodchem.2021.131969 35026473

[46] Zhu W Luan H Bu Y Li X Li J Ji G. Flavor characteristics of shrimp sauces with different fermentation and storage time. LWT. (2019) 110:142–51. doi: 10.1016/j.lwt.2019.04.091

[47] Maggi F Papa F Cristalli G Sagratini G Vittori S. Characterisation of the mushroom-like flavour of *Melittis melissophyllum* L. subsp. melissophyllum by headspace solid-phase microextraction (HS-SPME) coupled with gas chromatography (GC–FID) and gas chromatography–mass spectrometry (GC–MS). Food Chem. (2010) 123:983–92. doi: 10.1016/j.foodchem.2010.05.049

