# Supplementary material for: Effect of atmospheric cold plasma on the quality of fish meat from different parts of larger yellow croaker during refrigeration
Source: Front Nutr. 2026 Jan 27;13:1750328. doi: 10.3389/fnut.2026.1750328 (PMC12886045; doi:10.3389/fnut.2026.1750328)
Supplement: Supplementary file 1 [file Table_1.docx]

**Supplementary Table 1.** Changes in the percentage of T2 of large yellow croaker stored in ice at 4 ℃.

| **Storage times (d)** | **Group** | **0** | **2** | **4** | **6** | **8** |
| --- | --- | --- | --- | --- | --- | --- |
| P_21_/% | ACP belly | 3.263±1.340^aB^ | 3.251±1.047^aA^ | 3.044±0.219^aC^ | 1.829±0.267^aB^ | 1.776±0.013^aB^ |
|  | ACP back | 1.428±0.092^aA^ | 1.234±0.083^abB^ | 1.195±0.181^bA^ | 1.148±0.073^bA^ | 0.885±0.103^cA^ |
|  | Control belly | 2.272±0.320^aAB^ | 1.852±0.530^aC^ | 1.811±0.460^aB^ | 1.692±0.476^aB^ | 1.525±0.344^aB^ |
|  | Control back | 1.348±0.435^aA^ | 1.299±0.062^aD^ | 1.108±0.077^aA^ | 1.022±0.079^aA^ | 1.019±0.044^aA^ |
| P_22_/% | ACP belly | 95.083±1.498^aA^ | 94.458±0.052^aA^ | 93.905±0.749^aA^ | 93.566±2.875^aA^ | 92.814±0.889^aA^ |
|  | ACP back | 97.656±0.225^aBC^ | 97.806±1.376^aBC^ | 97.819±0.434^aC^ | 96.858±1.679^aAB^ | 96.479±0.831^aB^ |
|  | Control belly | 96.275±0.340^cAB^ | 96.376±0.811^cB^ | 95.337±0.282^bcB^ | 94.222±1.305^abAB^ | 93.079±0.984^aA^ |
|  | Control back | 98.015±0.739^aC^ | 97.953±0.070^aC^ | 97.744±0.778^aC^ | 97.726±0.124^aB^ | 97.407±1.686^aB^ |
| P_23_/% | ACP belly | 1.654±0.038^aA^ | 2.291±0.283^bC^ | 3.052±0.234^cB^ | 4.605±0.040^dB^ | 5.410±0.553^eC^ |
|  | ACP back | 0.906±0.131^aB^ | 0.960±0.150^aA^ | 0.986±0.097^aA^ | 1.994±0.825^bA^ | 2.636±0.047^bA^ |
|  | Control belly | 1.453±0.062^aC^ | 1.772±0.396^aB^ | 2.852±0.704^bB^ | 4.086±0.585^cB^ | 5.395±0.557^cC^ |
|  | Control back | 0.637±0.036^aD^ | 0.748±0.104^abA^ | 1.148±0.568^abcA^ | 1.252±0.162^bcA^ | 1.574±0.100^cB^ |

^a-e^ Represents within-group differences, ^A-D^ represents between-group differences.

**Supplementary Table 2** Changes in the fatty acid profile of different parts of fish with different treatments during the storage period (g/100g lipid).

|  | Control Belly | | | | | Control Back | | | | | ACP Belly | | | | | ACP Back | | | | |
| --- | --- | --- | --- | --- | --- | --- | --- | --- | --- | --- | --- | --- | --- | --- | --- | --- | --- | --- | --- | --- |
| Fatty acid | 0d | 2d | 4d | 6d | 8d | 0d | 2d | 4d | 6d | 8d | 0d | 2d | 4d | 6d | 8d | 0d | 2d | 4d | 6d | 8d |
| C12:0 | 0.0062±0.0002aA | 0.0013±0.0002bA | 0.0018±0.0001cA | 0.0025±0.0003dA | 0.0047±0.0002eA | 0.0016±0.0003aB | 0.0026±0.0003bB | 0.0017±0.0002aA | 0.0009±0.0001cB | 0.0023±0.0003bB | 0.0061±0.0002aA | 0.0045±0.0001bC | 0.0054±0.0000cB | 0.0058±0.0001dC | 0.0048±0.0002abC | 0.0012±0.0003aB | 0.0022±0.0002cD | 0.0017±0.0002bA | 0.0012±0.0002aB | 0.0014±0.0002abC |
| C13:0 | 0.0027±0.0004^aC^ | 0.0008±0.0001^cA^ | 0.0018±0.0001^cA^ | 0.0012±0.0002_bA_ | 0.0017±0.0003^aA^ | 0.001±0.0002^abA^ | 0.0017±0.0003^cBC^ | 0.0011±0.0002^abA^ | 0.0008±0.0001^aB^ | 0.0014±0.0002^bcB^ | 0.0019±0.0002^aB^ | 0.0020±0.0001^aC^ | 0.0024±0.0002^bB^ | 0.0028±0.0001^cC^ | 0.0023±0.0002^bA^ | 0.0005±0.0004^aA^ | 0.0015±0.0003^cB^ | 0.0012±0.0001^bcA^ | 0.0005±0.0000^aD^ | 0.0009±0.0003^abC^ |
| C14:0 | 0.3037±0.0050^aA^ | 0.0533±0.0041^bA^ | 0.0770±0.0102^cA^ | 0.1102±0.0147^dA^ | 0.2163±0.0095^eA^ | 0.0379±0.0074^aB^ | 0.1402±0.0053^cB^ | 0.0889±0.0066^bA^ | 0.0310±0.0106^aB^ | 0.0893±0.0078^bB^ | 0.2462±0.0027^aC^ | 0.2382±0.0013^abC^ | 0.2468±0.0019^aB^ | 0.2996±0.0010^cC^ | 0.2322±0.0097^bC^ | 0.0548±0.0087^aD^ | 0.0856±0.0009^cD^ | 0.0789±0.0023^cA^ | 0.0414±0.0059^aA^ | 0.0325±0.0024^aD^ |
| C15:0 | 0.0462±0.0024^aA^ | 0.0100±0.0012^bA^ | 0.0134±0.0011^cA^ | 0.0219±0.0013^dA^ | 0.0355±0.0022^eC^ | 0.0136±0.0029^aA^ | 0.0860±0.0010^aA^ | 0.0155±0.0011^aB^ | 0.0050±0.0006^aB^ | 0.0142±0.0023^aB^ | 0.0334±0.0022^aA^ | 0.0414±0.0010^cA^ | 0.0382±0.0005^bC^ | 0.0444±0.0018^dC^ | 0.0364±0.0017^bC^ | 0.0371±0.0476^aA^ | 0.0621±0.0796^aA^ | 0.0145±0.0005^aAB^ | 0.0076±0.0004^aD^ | 0.0061±0.0006^aA^ |
| C16:0 | 3.8753±0.1015^aA^ | 0.5876±0.0097^bA^ | 0.7753±0.0210^cA^ | 1.3993±0.0324^dA^ | 2.5479±0.0268^eA^ | 0.8666±0.0618^bA^ | 1.7013±0.0066^dB^ | 1.0636±0.0885^cB^ | 0.3834±0.0091^aB^ | 1.2238±0.2035^cB^ | 2.8312±0.1347^aB^ | 3.0031±0.0029^bC^ | 3.2018±0.0708^cC^ | 3.6883±0.0781^dC^ | 2.9366±0.0448^abC^ | 0.7925±0.0850^bA^ | 1.0035±0.0023^cD^ | 0.9680±0.0048^cB^ | 0.4914±0.0662^aD^ | 0.4159±0.0158^aD^ |
| C17:0 | 0.0609±0.0061^dC^ | 0.0130±0.0004^aA^ | 0.0166±0.0021^aA^ | 0.0273±0.0046^bB^ | 0.0489±0.0046^cA^ | 0.0152±0.0011^aA^ | 0.0230±0.0004^bA^ | 0.0189±0.0008^cAB^ | 0.0074±0.0011^dA^ | 0.0171±0.0006^eB^ | 0.0345±0.0021^aB^ | 0.1601±0.2060^aA^ | 0.0495±0.0040^aC^ | 0.0960±0.0082^aC^ | 0.0362±0.0057^aC^ | 0.0113±0.0015^bA^ | 0.0194±0.0006^cA^ | 0.0219±0.0018^cB^ | 0.0128±0.0021^bA^ | 0.0082±0.0006^aD^ |
| C18:0 | 0.9651±0.0193^aC^ | 0.1617±0.0072^bA^ | 0.2181±0.0193^cA^ | 0.3215±0.0223^dA^ | 0.6656±0.0074^eA^ | 0.2062±0.0105^aA^ | 0.2871±0.0074^bB^ | 0.2307±0.0064^cAB^ | 0.0974±0.0075^dB^ | 0.2821±0.0068^eB^ | 0.5456±0.0266^aB^ | 0.6639±0.0128^bC^ | 0.7896±0.0180^cC^ | 0.9232±0.0185^dC^ | 0.5792±0.0092^eC^ | 0.2006±0.0121^aA^ | 0.2659±0.0049^bD^ | 0.2467±0.0079^cB^ | 0.1339±0.0135^dD^ | 0.1023±0.0079^eD^ |
| C20:0 | 0.0427±0.0078^aA^ | 0.0089±0.0009^aA^ | 0.0528±0.0703^aA^ | 0.0177±0.0011^aA^ | 0.0342±0.0017^aA^ | 0.0115±0.0010^bC^ | 0.0185±0.0005^dB^ | 0.0117±0.0014^bA^ | 0.0043±0.0008^aB^ | 0.0147±0.0005^cB^ | 0.0288±0.0006^bB^ | 0.0258±0.0012^aC^ | 0.0392±0.0006^cA^ | 0.0467±0.0010^dC^ | 0.0302±0.0008^bC^ | 0.0087±0.0008^bC^ | 0.0131±0.0007^cD^ | 0.0156±0.0011^dA^ | 0.0084±0.0013^bD^ | 0.0052±0.0006^aD^ |
| C21:0 | 0.0118±0.0012^cA^ | 0.0022±0.0001^aA^ | 0.0022±0.0002^aA^ | 0.0029±0.0004^aA^ | 0.0055±0.0004^bC^ | 0.0019±0.0004^aB^ | 0.0036±0.0005^cB^ | 0.0026±0.0002^bA^ | 0.0022±0.0001^abA^ | 0.0026±0.0002^bA^ | 0.0028±0.0009^aB^ | 0.0077±0.0004^bC^ | 0.0140±0.0017^cB^ | 0.0084±0.0008^bB^ | 0.0046±0.0006^aB^ | 0.0016±0.0001^aB^ | 0.0028±0.0003^dA^ | 0.0025±0.0001^cdA^ | 0.0024±0.0002^bdA^ | 0.0021±0.0003^bA^ |
| C22:0 | 0.0263±0.0044^cA^ | 0.0073±0.0009^abA^ | 0.0048±0.0007^aA^ | 0.0079±0.0003^abB^ | 0.0088±0.0003^bB^ | 0.0109±0.0004^dB^ | 0.0080±0.0005^cAB^ | 0.0048±0.0006^bA^ | 0.0033±0.0004^aA^ | 0.0076±0.0008^cB^ | 0.0161±0.0004^aC^ | 0.0172±0.0006^aC^ | 0.0203±0.0012^bB^ | 0.0251±0.0004^cC^ | 0.0174±0.0012^aC^ | 0.0048±0.0009^bD^ | 0.0087±0.0005^dB^ | 0.0061±0.0007^cA^ | 0.0040±0.0005^bA^ | 0.0026±0.000^2aA^ |
| C23:0 | 0.0599±0.0012^aC^ | 0.0089±0.0003^bA^ | 0.0125±0.0012^cC^ | 0.0196±0.0009^dA^ | 0.0365±0.0019^eA^ | 0.0121±0.0030^bA^ | 0.0211±0.0007^cA^ | 0.0124±0.0012^bA^ | 0.0043±0.0005^aB^ | 0.0141±0.0003^bB^ | 0.0249±0.0025^aB^ | 0.0434±0.0024^dB^ | 0.0388±0.0005^cD^ | 0.0416±0.0007^cdC^ | 0.0297±0.0009^bC^ | 0.0101±0.0004^bA^ | 0.0147±0.0008^dA^ | 0.0124±0.0010^cB^ | 0.0074±0.0007^aD^ | 0.0062±0.0004^aD^ |
| C24:0 | 0.0222±0.0005^cA^ | 0.0048±0.0006^aA^ | 0.0068±0.0005^bA^ | 0.0078±0.0003^bA^ | 0.0316±0.0011^dA^ | 0.0051±0.0003^bB^ | 0.0080±0.0003^dB^ | 0.0064±0.0003^cA^ | 0.0027±0.0006^aC^ | 0.0058±0.0005^bB^ | 0.0161±0.0004^bC^ | 0.0135±0.0004^aC^ | 0.0156±0.0011^bB^ | 0.0194±0.0010^cC^ | 0.0137±0.0005^aC^ | 0.0082±0.0005^dD^ | 0.0067±0.0004^cD^ | 0.0075±0.0004^cdA^ | 0.0048±0.0006^bD^ | 0.0036±0.0006^aD^ |
| ∑SFA | 5.5119±0.1760^aC^ | 0.8567±0.0174^bA^ | 1.2291±0.1124^cA^ | 1.8543±0.0316^dB^ | 3.5791±0.0842^eA^ | 1.1640±0.0200^aA^ | 2.3322±0.0136^bB^ | 1.4088±0.01171^cB^ | 0.5749±0.0682^dA^ | 1.5770±0.0851^eB^ | 3.8328±0.0495^aB^ | 4.1205±0.0452^bC^ | 4.4518±0.1013^cC^ | 5.1921±0.1124^dC^ | 3.8332±0.0483^aC^ | 1.0870±0.0735^aA^ | 1.4659±0.0261^bD^ | 1.3648±0.0334^cAB^ | 0.6790±0.0303^dA^ | 0.5945±0.0153^eD^ |
| C14:1 | 0.0067±0.0009^dB^ | 0.0019±0.0004^aA^ | 0.0013±0.0002^aA^ | 0.0035±0.0004^bB^ | 0.0054±0.0005^cA^ | 0.0019±0.0004^bA^ | 0.0050±0.0006^dC^ | 0.0020±0.0003^bA^ | 0.0009±0.0003^aA^ | 0.0028±0.0004^cB^ | 0.0111±0.0009^dC^ | 0.0078±0.0003^bC^ | 0.0064±0.0006^aB^ | 0.0078±0.0006^bC^ | 0.0095±0.0010^cC^ | 0.0016±0.0004^cA^ | 0.0025±0.0003^dA^ | 0.0013±0.0002^bcA^ | 0.0011±0.0002^abA^ | 0.0008±0.0001^aD^ |
| C16:1 | 0.9769±0.0287^dB^ | 0.1394±0.0028^aA^ | 0.1887±0.0032^aA^ | 0.4731±0.0131^bB^ | 0.6752±0.0111^cA^ | 0.2629±0.0339^bA^ | 0.5659±0.0252^dB^ | 0.2929±0.0063^bA^ | 0.1234±0.0189^aA^ | 0.3136±0.0121^cB^ | 1.0391±0.0232^dC^ | 0.9704±0.0150^bC^ | 0.9099±0.0066^aB^ | 0.9436±0.0086^bC^ | 1.0139±0.0083^cC^ | 0.1790±0.0086^cA^ | 0.2596±0.0012^dA^ | 0.2457±0.0047^bcA^ | 0.1162±0.0051^abA^ | 0.1146±0.0014^aD^ |
| C20:1 | 0.1837±0.0016^aA^ | 0.0306±0.0010^bA^ | 0.0380±0.0017^cA^ | 0.0575±0.0049^dA^ | 0.0931±0.0006^eA^ | 0.0332±0.0012^aB^ | 1.6699±0.0152^bB^ | 0.7172±0.0090^cB^ | 0.3098±0.0090^dB^ | 1.0918±0.0080^eB^ | 2.5206±0.0337^aC^ | 2.8107±0.0363^bC^ | 3.0355±0.0050^cC^ | 3.5553±0.0127^dC^ | 2.4970±0.0048^aC^ | 0.6862±0.0044^aD^ | 0.8960±0.0053^bD^ | 0.8510±0.0113^cD^ | 0.3781±0.0051^dD^ | 0.3513±0.0014^eD^ |
| C22:1n9 | 0.0218±0.0013^aA^ | 0.0032±0.0005^bA^ | 0.0054±0.0004^cA^ | 0.0068±0.0004^dA^ | 0.0130±0.0004^eA^ | 0.0045±0.0004^aB^ | 0.0698±0.0014^bB^ | 0.0298±0.0016^cB^ | 0.0119±0.0007^dB^ | 0.0467±0.0039^eB^ | 0.1127±0.0006^aC^ | 0.1170±0.0002^cC^ | 0.1169±0.0003^cC^ | 0.1613±0.0011^dC^ | 0.1148±0.0014^bC^ | 0.0313±0.0011^aD^ | 0.0383±0.0003^bD^ | 0.0363±0.0009^cD^ | 0.0236±0.0006^dD^ | 0.0162±0.0004^eA^ |
| C18:1n9c | 3.6751±0.1278^aC^ | 0.4945±0.0074^bC^ | 0.6621±0.0233^cA^ | 1.1755±0.0097^dC^ | 2.4344±0.0048^eA^ | 0.7195±0.0066^aB^ | 0.0093±0.0004^bAB^ | 0.0036±0.0009^bB^ | 0.0083±0.0004^bA^ | 0.0072±0.0004^bB^ | 0.0116±0.0008^aA^ | 0.0153±0.0005^bB^ | 0.0159±0.0003^bB^ | 0.0199±0.0012^cB^ | 0.0132±0.0012^aC^ | 0.0031±0.0003^bA^ | 0.0053±0.0002^dA^ | 0.0054±0.0001^dB^ | 0.0041±0.0003^cA^ | 0.0025±0.0003^aD^ |
| C24:1 | 0.0828±0.0007^dA^ | 0.0302±0.0009^aA^ | 0.0285±0.0005^aA^ | 0.0549±0.0035^bA^ | 0.0776±0.0009^cA^ | 0.0292±0.0006^bB^ | 0.0322±0.0008^cB^ | 0.0305±0.0004^bcB^ | 0.0142±0.0009^aB^ | 0.0388±0.0021^dB^ | 0.0766±0.0009^cC^ | 0.0630±0.0010^aC^ | 0.0713±0.0009^bC^ | 0.0776±0.0007^cC^ | 0.0629±0.0006^aC^ | 0.0214±0.0004^bD^ | 0.0285±0.0007^dD^ | 0.0293±0.0007^dA^ | 0.0227±0.0005^cD^ | 0.0177±0.0005^aD^ |
| ∑MUFA | 5.0542±0.0431^aA^ | 0.7064±0.0127^bA^ | 0.9195±0.0039^cA^ | 1.7560±0.0045^dA^ | 3.2926±0.0080^eA^ | 1.0217±0.0017^aB^ | 2.3267±0.0139^bB^ | 1.0829±0.0094^cB^ | 0.4559±0.0069^dB^ | 1.4891±0.0201^eB^ | 3.7915±0.0224^aC^ | 4.0131±0.0211^bC^ | 4.1833±0.0218^cC^ | 4.7425±0.0130^dC^ | 3.6925±0.0117^eC^ | 0.9171±0.0137^aD^ | 1.2373±0.0146^bD^ | 1.1783±0.0063^cD^ | 0.5468±0.0056^dD^ | 0.5071±0.0047^eD^ |
| C18:2n6c | 2.0665±0.0078^aC^ | 0.2921±0.0041^bA^ | 0.3804±0.0048^cA^ | 0.4758±0.0284^dA^ | 0.9900±0.0030^eA^ | 0.3214±0.0171^aA^ | 0.6075±0.0056^bB^ | 0.1665±0.0029^cB^ | 0.1375±0.0029^dB^ | 0.5447±0.0044^eB^ | 0.9876±0.0061^aB^ | 1.0578±0.0359^bC^ | 1.4846±0.0214^cC^ | 2.0010±0.0008^dC^ | 1.0103±0.0050^aC^ | 0.3097±0.0048^aA^ | 0.4716±0.0034^bD^ | 0.4225±0.0088^cD^ | 0.2310±0.0059^dD^ | 0.1689±0.0063^eD^ |
| C18:3n6 | 0.0190±0.0023^cA^ | 0.0031±0.0003^aA^ | 0.0038±0.0001^aA^ | 0.0051±0.0005^aA^ | 0.0111±0.0005^bA^ | 0.0036±0.0005^aB^ | 0.0218±0.0015^bB^ | 0.0073±0.0005^cB^ | 0.0057±0.0004^dA^ | 0.0167±0.0005^eB^ | 0.0355±0.0006^aC^ | 0.0342±0.0017^aC^ | 0.0435±0.0012^bC^ | 0.0468±0.0005^cC^ | 0.0359±0.0005^aC^ | 0.0103±0.0006^bD^ | 0.0132±0.0006^cD^ | 0.0123±0.0007^cD^ | 0.0073±0.0003^aB^ | 0.0074±0.0001^aD^ |
| C18:3n3 | 0.2220±0.0014^aA^ | 0.0309±0.0003^bC^ | 0.0374±0.0012^cA^ | 0.0479±0.0003^dC^ | 0.1127±0.0008^eA^ | 0.0341±0.0011^dB^ | 0.0080±0.0007^cB^ | 0.0019±0.0004^aB^ | 0.0019±0.0005^aA^ | 0.0067±0.0006^bB^ | 0.0120±0.0014^bC^ | 0.0085±0.0004^aB^ | 0.0160±0.0003^cC^ | 0.0178±0.0005^dB^ | 0.0092±0.0007^aC^ | 0.0026±0.0004^aD^ | 0.0041±0.0004^bA^ | 0.0049±0.0003^cD^ | 0.0019±0.0002^dA^ | 0.0011±0.0002^eD^ |
| C20:2 | 0.0583±0.0016^aA^ | 0.0085±0.0009^bA^ | 0.0113±0.0008^cA^ | 0.0184±0.0009^dA^ | 0.0376±0.0013^eA^ | 0.0096±0.0007^aB^ | 0.0719±0.0004^dB^ | 0.0116±0.0008^bB^ | 0.0644±0.0034^aB^ | 0.1085±0.0029^cB^ | 0.1085±0.0077^aC^ | 0.1277±0.0077^bC^ | 0.1625±0.0046^cC^ | 0.2064±0.0047^dC^ | 0.1115±0.0018^aC^ | 0.0368±0.0024^aD^ | 0.0536±0.0043^bD^ | 0.0455±0.0043^cD^ | 0.0250±0.0049^dD^ | 0.0157±0.0006^eD^ |
| C20:3n6 | 0.0326±0.0017^dA^ | 0.0054±0.0005^aA^ | 0.0082±0.0005^bB^ | 0.0098±0.0007^bA^ | 0.0125±0.0007^cA^ | 0.0153±0.0006^aB^ | 0.0121±0.0010^bB^ | 0.0065±0.0006^cA^ | 0.0033±0.0006^dB^ | 0.0108±0.0006^eB^ | 0.0177±0.0007^aC^ | 0.0169±0.0002^aC^ | 0.0239±0.0013^bC^ | 0.0319±0.0007^cC^ | 0.0167±0.0005^aC^ | 0.0052±0.0005^bD^ | 0.0069±0.0002^cD^ | 0.0073±0.0005^cAB^ | 0.0051±0.0003^bD^ | 0.0033±0.0004^aD^ |
| C20:3n3 | 0.0124±0.0008^aC^ | 0.0026±0.0006^bA^ | 0.0046±0.0005^cA^ | 0.0068±0.0005^dA^ | 0.0090±0.0005^eA^ | 0.0046±0.0003^cB^ | 0.0059±0.0004^dB^ | 0.0016±0.0005^aB^ | 0.0037±0.0005^bB^ | 0.0052±0.0004^cdB^ | 0.0128±0.0008^cC^ | 0.0093±0.0004^bC^ | 0.0127±0.0006^cC^ | 0.0095±0.0003^bC^ | 0.0076±0.0004^aC^ | 0.0022±0.0004^aA^ | 0.0035±0.0003^bD^ | 0.0035±0.0003^bD^ | 0.0022±0.0003^aD^ | 0.0032±0.0003^bD^ |
| C20:4n6 | 0.1972±0.0059^aA^ | 0.0454±0.0037^bA^ | 0.0585±0.0011^cA^ | 0.0814±0.0011^dA^ | 0.1380±0.0026^eA^ | 0.0568±0.0037^bB^ | 0.0943±0.0032^dB^ | 0.0561±0.0016^bA^ | 0.0429±0.0026^aB^ | 0.0653±0.0019^cB^ | 0.1488±0.0013^aC^ | 0.1626±0.0007^bC^ | 0.1929±0.0035^cC^ | 0.1425±0.0026^dC^ | 0.1425±0.0026^eC^ | 0.0461±0.0030^bD^ | 0.0684±0.0008^dD^ | 0.0631±0.0017^cB^ | 0.0369±0.0006^aD^ | 0.0357±0.0006^aD^ |
| C20:5n3 | 0.3503±0.0024^aC^ | 0.0592±0.0004^bA^ | 0.0784±0.0008^dA^ | 0.1289±0.0092^dA^ | 0.2394±0.0014^eA^ | 0.0840±0.0023^aA^ | 0.1802±0.0045^bB^ | 0.1267±0.0028^cB^ | 0.0417±0.0031^dB^ | 0.1047±0.0054^eB^ | 0.2230±0.0127^aB^ | 0.2538±0.0025^bC^ | 0.2928±0.0032^cC^ | 0.3114±0.0062^dC^ | 0.2028±0.0045^eC^ | 0.0791±0.0022^bA^ | 0.1281±0.0019^cD^ | 0.1196±0.0091^cB^ | 0.0551±0.0053^aD^ | 0.0494±0.0016^aD^ |
| C22:6n3 | 0.9413±0.0154^aA^ | 0.2734±0.0044^bA^ | 0.3254±0.0050^cA^ | 0.5025±0.0060^dB^ | 0.7689±0.0034^eA^ | 0.3493±0.0032^bB^ | 0.5982±0.0042^dB^ | 0.4221±0.0041^cB^ | 0.2274±0.0176^aA^ | 0.3363±0.0032^bB^ | 0.7790±0.0004^aC^ | 0.7957±0.0079^bC^ | 0.8673±0.0074^cC^ | 0.8469±0.0036^dC^ | 0.7335±0.0129^eC^ | 0.2902±0.0012^aD^ | 0.4140±0.0097^bD^ | 0.3800±0.0020^cD^ | 0.2379±0.0074^dA^ | 0.2145±0.0033^eD^ |
| ∑PUFA | 3.9048±0.0081^aA^ | 0.7327±0.0096^bA^ | 0.9287±0.0172^cA^ | 1.2721±0.0186^dA^ | 2.3240±0.0163^eC^ | 0.8788±0.0122^bB^ | 1.6404±0.0702^dB^ | 0.8151±0.0167^bB^ | 0.4659±0.0143^aB^ | 1.2765±0.1360^cB^ | 2.2986±0.0026^aC^ | 2.4288±0.0051^bC^ | 3.0728±0.0703^cC^ | 3.6469±0.0227^dC^ | 2.2989±0.0047^aC^ | 0.7887±0.0072^aD^ | 1.1533±0.0077^bD^ | 1.0546±0.0231^cD^ | 0.5814±0.0067^dD^ | 0.4930±0.0023^eA^ |
| Total | 14.6881±0.3912^aC^ | 2.2877±0.0097^bA^ | 2.9453±0.0061^cA^ | 4.8890±0.0141^dA^ | 9.1907±0.0448^eA^ | 3.0755±0.0779^aA^ | 6.2659±0.0450^bB^ | 3.2962±0.0067^cB^ | 1.4438±0.0252^dB^ | 4.2969±0.1247^eB^ | 9.9378±0.0129^aB^ | 10.5319±0.0216^bC^ | 11.5611±0.0215^cC^ | 13.5482±0.0138^dC^ | 9.9601±0.1232^aC^ | 2.7678±0.0325^aA^ | 3.8599±0.0476^bD^ | 3.6116±0.0082^cD^ | 1.7993±0.0349^dD^ | 1.5712±0.0314^eD^ |
